# [^18^F]FET PET enables histopathology-validated detection of non-contrast-enhancing glioblastoma

**DOI:** 10.1038/s41467-026-78980-1

**Published:** 2026-11-06

**Authors:** R. Stürzl, S. V. Kirchleitner, J. Lorenz, K. J. Schmidbauer, V. Stöcklein, S. Quach, L. Kaiser, S. Stöcklein, A. J. Zounek, E. Barci, I. von Polenz, J. Blobner, T. Greve, M. Brendel, P. Harter, V. C. Ruf, R. Rupprecht, F. Ringel, D. Kalasauskas, M. J. Riemenschneider, N. Thon, L. von Baumgarten, J-C Tonn, N. L. Albert

**Affiliations:** 1 Department of Nuclear Medicine, LMU University Hospital, LMU Medizin, Ludwig-Maximilians-Universität München, Munich, Germany; 2 Department of Psychiatry and Psychotherapy, University Regensburg, Regensburg, Germany; 3 Department of Neurosurgery, LMU University Hospital, LMU Medizin, Ludwig-Maximilians-Universität München, Munich, Germany; 4 Heidelberg University, Department of Neurosurgery, Heidelberg, Germany; 5 Department of Neuropathology, Regensburg University Hospital, Regensburg, Germany; 6 Department of Neurology, LMU University Hospital, LMU Medizin, Ludwig-Maximilians-Universität München, Munich, Germany; 7 Department of Neurosurgery, University Hospital OWL, Bielefeld, Germany; 8 Department of Radiology, LMU University Hospital, LMU Medizin, Ludwig-Maximilians-Universität München, Munich, Germany; 9 Bavarian Cancer Research Center (BZKF), LMU Partner Site Munich, Munich, Germany; 10 German Center for Neurodegenerative Diseases (DZNE), Munich, Germany; 11 Munich Cluster for Systems Neurology (SyNergy), Munich, Germany; 12 Institute of Neuropathology, LMU Medizin, Ludwig-Maximilians-Universität München, Munich, Germany; 13 Department of Neurosurgery, Knappschaft University Hospital Bochum, Bochum, Germany; 14 German Cancer Consortium (DKTK), Partner Site Munich, German Cancer Research Center (DKFZ), Heidelberg, Germany

**Keywords:** CNS cancer, Cancer imaging, Molecular medicine, Cancer

## Abstract

Accurate visualization of glioblastoma is essential for guiding biopsy, resection and radiotherapy. Contrast-enhanced MRI is routine in clinical practice, yet it can miss metabolically active tumor outside areas of blood-brain barrier disruption. In a histopathology-validated cohort with 472 tissue samples of 61 patients with IDH-wildtype glioblastoma, we show that O-(2-[¹⁸F]fluoroethyl)-L-tyrosine PET ([^18^F]FET PET) depicts tumor tissue significantly better than contrast-enhanced MRI (CE-MRI), especially in regions that show no enhancement on MRI. [^18^F]FET PET achieves substantially higher sensitivity for tumor detection (91.2% vs 52.6%) and maintains high reliability in non-enhancing tissue (positive predictive value 98.3%), findings that are consistent across newly diagnosed and recurrent disease and in both cohorts undergoing either resection or biopsy. These results indicate that [^18^F]FET PET complements MRI by revealing MRI-occult tumor burden. Incorporating amino acid PET into routine planning may therefore improve the accuracy of tumor visualization and, ultimately, patient management.

## Abstract (web summary)

Contrast-enhanced magnetic resonance imaging (CE-MRI) is standard for patients with glioblastoma, but its ability to detect metabolically active tumours cells at the infiltrative edge is limited. Here, the authors present a prospective biopsy-validated study demonstrating that ¹⁸F-FET PET imaging can detect tumour tissue beyond that detected by CE-MRI in patients with IDH-wildtype glioblastoma.

## Introduction

Glioblastoma, isocitrate dehydrogenase (IDH) wildtype (WHO grade 4), is the most common primary malignant brain tumor in adults, characterized by rapid progression and dismal prognosis despite multimodal treatment strategies^1^. The extent of resection is one of the most important prognostic factors, with maximal safe resection being associated with significant improvements in both overall survival and progression-free survival^2–7^. Accurate preoperative visualization of vital tumor tissue is therefore essential to optimize surgical outcomes and subsequent adjuvant therapies.

Contrast-enhanced magnetic resonance imaging (CE-MRI) is the current standard for assessing tumor extent, offering high spatial resolution and detailed anatomical information^1,8^. However, CE-MRI relies on blood-brain barrier disruption for lesion visualization, potentially missing infiltrative tumor components without contrast enhancement^9,10^. Despite advances in MRI, reliable visualization of metabolically active but non-contrast-enhancing tumor remains an unresolved challenge in glioblastoma surgery and radiotherapy planning. In addition, therapy-related changes such as pseudoprogression and radiation necrosis may mimic tumor progression, thereby complicating interpretation and potentially leading to suboptimal treatment decisions^11,12^.

O-(2-[^18^F]fluoroethyl)-L-tyrosine positron emission tomography is a complementary imaging modality that was long thought to report mainly on amino-acid transport mediated by the L-type amino acid transporter LAT1, which is frequently overexpressed in glioma cells^13^. Emerging evidence, however, indicates that the [^18^F]FET signal reflects a composite of the activity of multiple amino acid transporters, together with tumor perfusion, microvascular architecture and blood-brain barrier properties, rather than LAT1 expression alone^14,15^. Several studies have demonstrated that [^18^F]FET PET can detect metabolically active tumor tissue, particularly in infiltrative margins, more sensitively than CE-MRI^16,17^. Although several studies have reported larger tumor volumes on amino acid PET than on contrast-enhanced MRI in glioma patients^16,18,19^, histopathology-verified data on the spatial relationship between PET- and MRI-defined tumor borders remain scarce and are largely confined to small, clinically heterogeneous biopsy-controlled series with limited numbers of sampling locations^16,20,21^.

Here, we show that [^18^F]FET PET detects tumor tissue beyond CE-MRI in a cohort exclusively comprising patients with glioblastoma, IDH-wildtype (WHO grade 4), defined according to the 2021 WHO classification of tumors of the central nervous system^22^. The analysis is based on 472 spatially matched, histopathology-verified tissue samples from stereotactic biopsies and resections. In particular, [^18^F]FET PET-positive, non-enhancing samples have a PPV of 98.3% for histologically confirmed tumor, indicating the value of [^18^F]FET PET for detecting metabolically active tumor tissue that would be missed by MRI alone.

## Results

### Baseline characteristics

**Fig. 1:**
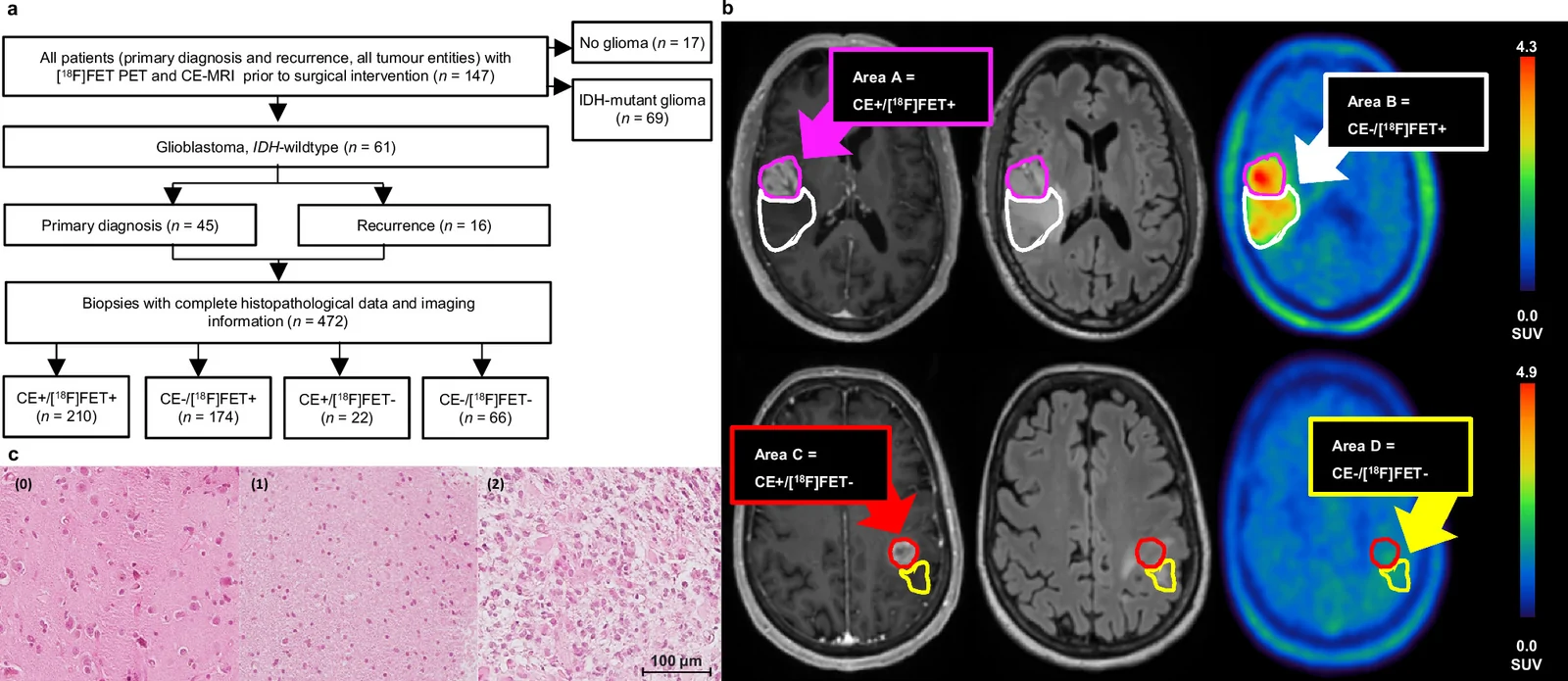
Patient inclusion, imaging-defined area categories and histopathological classification. **a** Flowchart illustrating patient inclusion, biopsy sampling and distribution per area. **b** Representative depiction of the four area categories (A-D) with colour-coded contours. From left to right: contrast-enhanced T1-weighted magnetic resonance imaging (CE-MRI), T2-weighted/fluid-attenuated inversion recovery (T2/FLAIR) MRI and O-(2-[¹⁸F]fluoroethyl)-L-tyrosine positron emission tomography ([^18^F]FET PET). Upper row: Area A (purple contour) denotes regions positive on [^18^F]FET PET (maximum tumor-to-background ratio (TBRmax) ≥ 1.6) with corresponding contrast enhancement; Area B (white contour) denotes regions positive on [^18^F]FET PET without contrast enhancement; Lower row: Area C (red contour) shows contrast enhancement without PET-positivity (TBRmax <1.6); Area D (yellow contour) shows neither contrast enhancement nor PET-positivity. **c** Representative hematoxylin and eosin (H&E) stainings from one patient illustrating the three histological categories used in this study (100× magnification). (0) No tumor: Cortex and/or white matter with preserved normal architecture and without clear evidence of neoplastic glial cells; only mild reactive changes may be present. (1) Infiltration zone: Moderately increased cellularity with a visible mixture of tumor and non-neoplastic elements; cortical neurons remain identifiable and are often surrounded by small clusters of atypical glial cells, while the overall parenchymal structure is largely maintained and features of tumor core are absent. (2) Solid tumor: Densely packed glioblastoma with almost complete loss of normal cortical or white-matter architecture and only rare residual non-neoplastic cells; tumor forms confluent, pleomorphic proliferations with mitotic figures and typically microvascular proliferation and necrosis. Scale bar: 100 µm. IDH isocitrate dehydrogenase. Source data are provided as a Source data file.

A total of 61 patients with glioblastoma, IDH-wildtype (WHO CNS classification 2021) were included. All patients had undergone [^18^F]FET PET and CE-MRI prior to surgery. 45 patients (73.8%) had newly diagnosed disease and 16 patients (26.2%) presented with recurrence (Fig. 1a). Positive [^18^F]FET uptake, defined as regions exhibiting a standard uptake value (SUV) ≥ 1.6 times the mean background activity (tumor-to-background ratio/TBR ≥ 1.6), was observed in all patients (100%), whereas CE on MRI was present in 56/61 cases (91.8%). In all CE-positive cases, [^18^F]FET uptake extended beyond the enhancing regions. Tissue acquisition was performed via stereotactic biopsy in 26 patients (42.6%) and in conjunction with partial or gross total resection in 35 patients (57.4%). Median age at the time of surgical intervention was 59 years (range 33–84), and median interval between PET and MRI 1 day (range 0–28). A subgroup analysis was further conducted only including patients with a maximum interval of 14 days between PET and MRI, and findings were concordant with the main analysis without clinically relevant differences. All baseline characteristics of the overall cohort are summarized in Table 1.

**Table 1:** Baseline characteristics of the cohort

Characteristic		
Age (years; median, range)		59, 33–84
Time interval between [^18^F]FET PET and CE-MRI (days; median, range)		1, 0–28
Time interval between imaging and surgical intervention (days; median, range)		2, 0–26
Obtained samples per patient (*n*; median, range)		5, 1–27
Sex (*n*, %)	Female	26, 42.6
	Male	35, 57.4
Diagnosis status (*n*, %)	Newly diagnosed	45, 73.8
	Recurrent disease	16, 26.2
Surgical intervention (*n*, %)	Stereotactic biopsy	26, 42.6
	Resection	35, 57.4
[^18^F]FET-positivity (*n*, %)	Yes (TBR_max_ ≥ 1.6)	61, 100.0
Contrast enhancement (*n*, %)	Yes	56, 91.8
	No	5, 8.2

A total of 472 samples were obtained (median per patient: 5; range: 1–27). While all samples had definitive histology and imaging data, quantitative imaging information could not be obtained for 8 biopsies due to intraoperative technical issues. Of all samples, 210 (44.5%) were obtained from CE+/[^18^F]FET+ regions (defined as area A) and 174 (36.9%) from CE−/[^18^F]FET+ regions (defined as area B). In addition, CE+/[^18^F]FET- regions were defined as area C (*n* = 22; 4.6%) and CE-/[^18^F]FET- regions as area D (*n* = 66; 14.0%). The exact distribution across imaging-defined areas A-D, including the binary coding of T2/FLAIR abnormality (present vs. absent), is shown in Table 2 for both histological categories, solid tumor and infiltration zone. The same distribution is additionally provided separately for the initial diagnosis and recurrence cohorts in Supplementary Tables S1A and S1B. Regarding imaging information, both SUV and TBR (mean and maximum) were consistently higher in samples with histologically confirmed tumor than in samples without histological evidence of tumor (*p* < 0.001). Among the spatially defined areas, the highest uptake values were observed in area A ([^18^F]FET+/CE+), followed by area B ([^18^F]FET+/CE−), whereas areas C and D (both [^18^F]FET-) showed significantly lower values (TBR_max_: 2.83 in area A vs 2.22 in area B vs 1.49 in area C and 1.34 in area D; *p* < 0.001). Exact descriptive statistics of median SUV_max_, SUV_mean_, TBR_mean_, and TBR_max_ stratified by tumor-positive versus tumor-negative samples and by area type as well as overall are shown in Table 3.

**Table 2:** Tissue sample distribution

	No tumor	Infiltration zone	Solid tumor	Overall	
	(*n*, %)	(*n*, %)	(*n*, %)	(*n*, %)	
**A0**	4 (6.3)	6 (3.1)	22 (10.1)	32 (6.8)	210 (44.5)
**A1**	4 (6.3)	65 (33.9)	109 (50.2)	178 (37.7)	
**B0**	2 (3.2)	17 (8.9)	2 (0.9)	21 (4.4)	174 (36.9)
**B1**	1 (1.6)	70 (36.5)	82 (37.8)	153 (32.4)	
**C0**	1 (1.6)	7 (3.6)	0 (0.0)	8 (1.7)	22 (4.6)
**C1**	8 (12.7)	5 (2.6)	1 (0.5)	14 (3.0)	
**D0**	14 (22.2)	6 (3.1)	0 (0.0)	20 (4.2)	66 (14.0)
**D1**	29 (46.0)	16 (8.3)	1 (0.5)	46 (9.7)	
**Overall**	63 (100.0)	192 (100.0)	217 (100.0)	472 (100.0)	

Exact distribution across area types A-D, including binary coding for the absence (0) or presence (1) of T2/FLAIR alteration, divided according to histological strata and overall.

**Table 3:** Descriptive statistics for quantitative PET imaging metrics

	Tumor-pos.	Tumor-neg.	Area A	Area B	Area C	Area D	Overall
**SUV**_**mean**_	2.31 ± 0.97	1.25 ± 0.63	2.52 ± 0.93	2.21 ± 1.00	1.35 ± 0.21	1.22 ± 0.51	2.18 ± 1.01
**SUV**_**max**_	2.49 ± 1.04	1.39 ± 0.71	2.75 ± 1.02	2.39 ± 1.02	1.40 ± 0.15	1.39 ± 0.52	2.38 ± 1.10
**TBR**_**mean**_	2.20 ± 0.89	1.25 ± 0.57	2.59 ± 0.93	2.06 ± 0.68	1.24 ± 0.14	1.23 ± 0.25	2.11 ± 0.93
**TBR**_**max**_	2.41 ± 0.98	1.33 ± 0.62	2.83 ± 1.01	2.22 ± 0.68	1.49 ± 0.16	1.34 ± 0.26	2.34 ± 1.03

Median ( ± SD) values of standard uptake value (SUV_mean_, SUV_max_) and tumor-to-background ratio (TBR_mean_, TBR_max_) are shown for tumor-positive and tumor-negative samples, stratified by area type A-D and overall.

### Overall diagnostic performance: [^18^F]FET PET vs CE-MRI

To validate the TBR_max_ threshold used for defining [^18^F]FET-positivity, a ROC analysis was performed, demonstrating excellent discriminative performance for distinguishing non-tumor from tumor tissue (AUC = 0.908). The maximum Youden index identified a data-driven cut-off of 1.65, closely aligning with the literature-based threshold of 1.6 used in this study.

Applying this validated threshold, [^18^F]FET PET was superior or at least comparable to CE-MRI with respect to both tumor detection and exclusion. [^18^F]FET PET achieved a significantly higher sensitivity (91.2% vs 52.6%; *p* < 0.001) with comparable specificity (82.5% vs 73.0%; *p* = 0.146). For the positive predictive value (PPV), [^18^F]FET PET also performed significantly better (97.1% vs 92.7%; *p* = 0.015). Considered separately, the negative predictive value (NPV) for [^18^F]FET PET was likewise significantly higher (59.1% vs 19.2%; *p* < 0.001), underscoring [^18^F]FET PET’s superior ability to rule out tumor when findings are negative.

### [^18^F]FET PET reliably visualizes vital tumor beyond the contrast-enhancing margin

Beyond the overall diagnostic advantage, these findings are clinically meaningful particular with regards to CE-MRI-negative regions where [^18^F]FET PET reliably identifies tumor that would be classified as non-tumor-suspicious on contrast-enhanced imaging alone. This is supported by a high PPV of 98.3% across 174 biopsies from Area B (CE−/[^18^F]FET+). In practical terms, [^18^F]FET-positivity (TBR ≥ 1.6) in regions deemed non-suspicious by CE-MRI alone was highly associated with histologically confirmed tumor. Among CE-negative samples (B vs D), PPV’s differed significantly between [^18^F]FET-positive (Area B) and [^18^F]FET-negative regions (Area D, PPV 34.8%; *p* < 0.001), highlighting the selectivity of the [^18^F]FET signal for vital tumor. Figure 1b illustrates this pattern in a representative patient: increased [^18^F]FET uptake extends beyond the contrast-enhancing boundary, marking additional metabolically active tumor that would have remained undetected on CE-MRI alone. Consistent with this observation, 69.6% of CE-positive cases (39/56) showed [^18^F]FET-positivity ≥10 mm beyond the CE margin, quantitatively demonstrating the spatial expansion of tumor visualization achieved with [^18^F]FET PET. Exact values for sensitivity, specificity, PPV, and NPV—by imaging modality, area type, and pooled imaging definitions—are reported in Tables 4–6.

**Table 4:** Diagnostic performance of the three imaging modalities

	CE-MRI	T2/FLAIR	[^18^F]FET PET
**Sensitivity (%)**	52.6	85.3	91.2
**Specificity (%)**	73.0	33.3	82.5
**PPV (%)**	92.7	89.3	97.1
**NPV (%)**	19.2	25.9	59.1

Sensitivity, specificity, positive predictive value (PPV), and negative predictive value (NPV) for detecting histology-verified tumor; values are percentages.

**Table 5:** Diagnostic performance by composite CE-MRI/[18 F]FET PET-defined areas

	Area A (CE + /[^18^F]FET + )	Area B (CE-/[^18^F]FET + )	Area C (CE + /[^18^F]FET−)	Area D (CE−/[^18^F]FET−)
**Sensitivity (%, 95% CI)**	49.4 (44.6–54.2)	41.8 (37.1–46.6)	3.2 (1.9–5.4)	5.6 (3.8–8.3)
**Specificity (%, 95% CI)**	87.3 (76.9–93.4)	95.2 (86.9–98.4)	85.7 (75.0–92.3)	31.7 (21.6–44.0)
**PPV (%, 95% CI)**	96.2 (92.7–98.1)	98.3 (95.1–99.4)	59.1 (38.7–76.7)	34.8 (24.5–46.9)
**NPV (%, 95% CI)**	21.0 (16.5–26.3)	20.1 (16.0–25.1)	12.0 (9.3–15.3)	4.9 (3.2–7.5)

Sensitivity, specificity, positive predictive value (PPV), and negative predictive value (NPV) are reported as percentages with 95% confidence intervals for detecting histology-verified tumor across areas A-D.

**Table 6:** Diagnostic performance of CE-MRI, [^18^F]FET PET and their combined use

Imaging test definition	Sensitivity	Specificity	PPV	NPV
**CE**+	52.6%	73.0%	92.7%	19.2%
**[**^**18**^**F]FET**+	91.2%	82.5%	97.1%	59.1%
**CE+ and/or [**^**18**^**F]FET**+	94.4%	68.3%	95.1%	65.2%

Sensitivity, specificity, positive predictive value (PPV), and negative predictive value (NPV) for detecting histopathology-verified tumor. For pooled analyses, CE-positivity comprised groups A + C, [^18^F]FET-positivity groups A + B, and combined positivity groups A − C; values are percentages.

An exploratory subgroup analysis was performed for the five patients without CE on MRI. All five cases were [^18^F]FET-positive at patient level. From these five cases, 45 samples were available, including 30 from area B (CE−/[^18^F]FET+) and 15 from area D (CE−/[^18^F]FET−). All 30 area-B specimens were histologically tumor-positive, corresponding to a positive predictive value of 100%. This was comparable to the PPV of area B in the overall cohort (98.3%), with no significant difference when compared with the remainder of the whole cohort (30/30 vs 141/144; Fisher’s exact *p* = 1.00). Thus, even in the complete absence of contrast enhancement, [^18^F]FET-positivity remained highly predictive of histologically confirmed tumor. The molecular profile of these five cases showed no obvious pattern: MGMT promoter methylation was present in four cases, TERT mutation in three, LOH 1p/19q was absent in all cases, and CDKN2A/B alteration was detected in one case, while four cases lacked CDKN2A/B data. These findings support the robustness of [^18^F]FET-positivity for visualization of viable tumor in non-enhancing glioblastoma.

### Diagnostic performance and role of T2/FLAIR imaging

T2/FLAIR abnormalities are widely used to depict edema and tumor infiltration but are inherently non-specific, particularly in glioblastoma. This limitation is amplified in the recurrence setting after resection and radiochemotherapy, where gliosis frequently appears T2/FLAIR-positive and can mimic viable tumor tissue.

In our cohort [^18^F]FET PET achieved superior sensitivity (*p* = 0.006) and substantially higher specificity (*p* < 0.001) compared with T2/FLAIR. This specificity gap translated into a higher PPV for [^18^F]FET PET (*p* < 0.001) and a higher NPV (*p* < 0.001) compared with T2/FLAIR. Exact values for all four metrics (sensitivity, specificity, PPV, NPV) are provided in Table 4.

Of particular clinical relevance, T2/FLAIR was not reliable for identifying tumor in CE-negative tissue. Restricting the analysis to T2/FLAIR-positive samples that were CE-negative and [^18^F]FET-negative (T2/FLAIR+/CE−/[^18^F]FET− = area D1), the PPV for T2/FLAIR was 37.0% (17/46). By contrast, in the same CE-negative context, [^18^F]FET-positive specimens (Area B) yielded a PPV of 98.3% (171/174; *p* < 0.001) as stated before. Together, these data indicate that while T2/FLAIR-positivity is sensitive, its low specificity as well as its poor PPV in non-enhancing and [^18^F]FET-negative tissue limits its value for identifying additional tumor-positive areas, whereas [^18^F]FET PET provides reliable positive calls precisely in these regions.

### Robustness of [^18^F]FET PET across clinical and histological categories

Across disease stage, sampling method, and cell-density categories, [^18^F]FET PET maintained a consistently high positive predictive performance. In both newly diagnosed and recurrent glioblastoma, [^18^F]FET-positive samples inside and outside the contrast-enhancing margin (Areas A and B) showed similarly high PPV, with no meaningful difference (*p* = 0.624 in initial diagnosis; *p* = 0.344 in recurrence). In the newly diagnosed cohort, PPV in CE-/[^18^F]FET- tissue (Area D) was substantially lower than in Area B (*p* < 0.001), and pooling A + B versus D confirmed this gap (*p* < 0.001). In the recurrence subgroup, no biopsies were obtained from Area D; consequently, the B versus D and A + B versus D comparisons could not be performed.

The same pattern was observed when stratifying by sampling method. In specimens obtained during resection and in stereotactic biopsies, areas A and B yielded equally high PPV (A vs B: *p* = 1.00 in resections; *p* = 0.335 in stereotactic biopsies), whereas area D remained significantly inferior (*p* < 0.001). These findings indicate that the reliability of a positive [^18^F]FET signal does not depend on how tissue was obtained (exact values in Table 7).

**Table 7:** Diagnostic performance in prespecified subgroups

	Diagnostic metric	Area A (CE + /[^18^F]FET + )	Area B (CE−/[^18^F]FET + )	Area C (CE + /[^18^F]FET−)	Area D (CE−/[^18^F]FET−)
**Initial diagnosis (*n*** = **377)**	**Sensitivity**	42.7	46.4	3.7	7.1
	**Specificity**	98.1	94.4	87.0	20.4
	**PPV**	99.3	98.0	63.2	34.8
	**NPV**	22.3	22.8	13.1	3.5
**Recurrence (*n*** = **95)**	**Sensitivity**	74.4	24.4	1.2	n.a.
	**Specificity**	22.2	100.0	77.8	n.a.
	**PPV**	90.1	100.0	33.3	n.a.
	**NPV**	8.3	12.2	7.6	n.a.
**Stereotactic biopsy (*n*** = **349)**	**Sensitivity**	44.1	45.4	3.4	7.1
	**Specificity**	87.0	94.4	90.7	27.8
	**PPV**	94.9	97.8	66.7	35.0
	**NPV**	22.2	24.1	14.7	5.2
**Resection (*n*** = **123)**	**Sensitivity**	63.2	32.5	2.6	1.8
	**Specificity**	88.9	100.0	55.6	55.6
	**PPV**	98.6	100.0	42.9	33.3
	**NPV**	16.0	10.5	4.3	4.3

Sensitivity, specificity, positive predictive value (PPV), and negative predictive value (NPV) are reported as percentages for detecting histology-verified tumor, stratified by sampling method (stereotactic biopsy vs surgical resection) and disease stage (newly diagnosed vs recurrent).

Within tumor-positive samples (*n* = 409), [^18^F]FET PET outperformed CE-MRI in both tumor cell-density categories. Within the infiltration zone, detection with [^18^F]FET PET was 82.3% (158/192) versus 43.2% (83/192) with CE-MRI; in solid tumor, the rates were 99.1% (215/217) versus 60.8% (132/217), respectively. Paired analyses restricted to histology-positive samples confirmed the same pattern, with [^18^F]FET PET identifying tumor more frequently than CE-MRI in both strata (*p* < 0.001 for both), indicating superior detection, even in areas of lower cell density like the infiltration zone. Within each modality, detection was higher in solid tumor than in the infiltration zone ([^18^F]FET PET: 99.1% vs 82.3%; CE-MRI: 60.8% vs 43.2%; both *p* < 0.001). Consistent with this pattern, TBRmax correlated positively with semiquantitative histopathological tumor burden (Spearman *ρ* = 0.58, *p* < 0.001) and showed a significant increase in PET signal from infiltration zone to solid tumor (*p* < 0.001). Taken together, these findings indicate that while both modalities favor solid components, [^18^F]FET PET retains a clear advantage in each histologic stratum.

## Discussion

Within this prospectively enrolled, exploratory study with histological validation, we demonstrate that [^18^F]FET PET visualizes glioblastoma tissue with significantly higher diagnostic accuracy than contrast-enhanced MRI. Overall, 472 samples were spatially correlated with CE-MRI and [^18^F]FET PET and analyzed histologically. Samples were obtained from 61 patients with glioblastoma, IDH-wildtype, classified according to the 2021 WHO CNS criteria^22^, including newly diagnosed and recurrent cases. In almost 70% of patients, [^18^F]FET-positive signal was detected at least 10 mm beyond the enhancing lesion border. In particular, [^18^F]FET-positivity in non-enhancing tissue corresponded to viable glioblastoma in nearly all samples (PPV 98.3%), indicating that [^18^F]FET PET reliably detects biologically active tumor beyond contrast enhancement.

Previous studies have reported only modest spatial congruence between contrast enhancement on MRI and amino acid PET uptake in high-grade glioma, with PET-defined tumor volumes frequently extending beyond the enhancing lesion^17–19^. Biopsy-validated PET/MRI studies reached conclusions congruent with ours, showing that PET-positive, non-enhancing regions commonly harbor tumor on histopathology^16,20^. Building on this work, our large cohort with histopathological confirmation strengthens the evidence that viable glioblastoma can be visualized by amino-acid PET beyond contrast enhancement on MRI.

An additional aspect not addressed in the present analysis is the potential contribution of dynamic [^18^F]FET PET. Dynamic parameters such as time–activity-curve shape and time-to-peak may provide complementary information on tracer kinetics and tumor biology and have been linked to glioma characterization, prognosis, and the identification of biologically more aggressive tumor subregions^15,23–25^. Moreover, biopsy-oriented dual-time-point approaches suggest that dynamic amino acid PET analyses may help refine the delineation of infiltrative tumor, including tissue beyond contrast enhancement and within MRI-abnormal regions^10,21,26^. However, compared with static assessment according to PET RANO 1.0 criteria, the role of dynamic metrics for biopsy-level characterization of tumor infiltration remains less well standardized^23^. Since dynamic imaging was available only in a subset of our cohort, these parameters were beyond the scope of the present study but may provide additional insight into the spatial mismatch between [^18^F]FET uptake and contrast enhancement.

This mismatch is clinically relevant because standards for local therapy remain largely defined by contrast enhancement, and glioblastoma typically recurs at the resection margin or along the high-dose radiation border^27,28^. Therefore, and because postoperative residual tumor burden is an established adverse prognostic factor, neurosurgical oncology has increasingly moved towards the concept of supramarginal resection, motivated by evidence that more extensive removal of infiltrating tumor may improve survival^2,3,6,7,29–34^. However, supramarginal resection is inconsistently defined, ranging from arbitrary extensions beyond enhancement to “FLAIRectomy”^35^. Given the non-specificity of T2/FLAIR hyperintensity^36^ and the risk of resecting eloquent but non-tumorous tissue^1^, our data underscore its limited reliability (PPV 37.0% in T2/FLAIR+ but CE− and [^18^F]FET− samples). By contrast, [^18^F]FET-positivity showed the high PPV reported above, indicating that [^18^F]FET PET provides a biologically specific map of tumor tissue and may enable individualized, PET-based supramarginal resection.

Radiotherapy target volumes likewise remain anchored to the resection cavity and residual enhancement, although amino acid PET can extend target definition beyond CE-MRI^37–39^. While we did not perform a specimen-wise distance analysis to the nearest contrast-enhancing lesion, we specifically addressed this issue at the patient level by assessing the spatial extent of [^18^F]FET-positivity beyond CE-MRI. In this analysis, [^18^F]FET-positivity frequently extended more than 1 cm beyond CE on MRI, with the corresponding [^18^F]FET+/CE− area showing a very high PPV for viable tumor, as outlined above. Thus, our findings support the relevance of [^18^F]FET PET for target definition beyond delineation by CE-MRI alone, even though this does not by itself establish a clinical benefit. The recently published GLIAA trial reported no outcome advantage for [^18^F]FET PET-based vs CE-MRI-based target delineation in stereotactic re-irradiation of recurrent glioblastoma^40^, this result should be interpreted cautiously, as the clinical efficacy of re-irradiation itself is being prospectively evaluated in the ongoing EORTC 2227-LEGATO trial (lomustine ± re-irradiation)^41^. Nonetheless, [^18^F]FET PET may still inform postoperative target visualization beyond contrast enhancement.

Given our histopathology-verified findings, monitoring disease course with a readout less dependent on contrast enhancement is biologically reasonable. RANO 2.0, while improving standardization, remains MRI-based and vulnerable to treatment-related blood-brain barrier effects that can decouple changes of contrast enhancement from true tumor dynamics^42,43^. In comparison, PET RANO 1.0 provides a harmonized framework for amino acid PET measurability and response categorization to capture metabolically active tumor beyond enhancement^44^. In glioblastoma, “measurable disease” was identified substantially more often on PET according to PET RANO 1.0 criteria than on MRI based on RANO 2.0, which would enable longitudinal assessment of objective responses in twice as many cases based on PET compared to MRI^45^. Early clinical analyses further suggest improved outcome discrimination with PET RANO 1.0, supporting PET-based response assessment as a more reliable approach for tracking tumor course^46^.

Despite its biological selectivity, amino acid PET has limitations: although [^18^F]FET-positivity was highly sensitive for viable tumor, the absence of [^18^F]FET signal did not exclude tumor (NPV 59.1%). This limitation is most pronounced in low-density infiltration, as [^18^F]FET PET identified solid tumor more often than the infiltration zone (99.1% versus 82.3%, *p* < 0.001), suggesting that tumor below a cellularity threshold may fall beneath detection. Consistent with this, TBRmax was positively associated with semiquantitative histopathological tumor burden and showed a significant stepwise increase across the categories no tumor, infiltration zone, and solid tumor. Defining how actual tumor cell density relates to quantitative PET metrics could support more biologically informed PET-based targeting for local therapy planning.

Methodologically, [^18^F]FET− regions (areas C and D) were under-sampled in this targeted sampling study, as additional specimens in apparently inactive tissue carry procedural risks. This limits conclusions regarding the extent to which PET-negativity reflects tumor-negative tissue in general and, in the presence of contrast enhancement, reliably indicates non-tumoral blood-brain barrier disruption, such as radiation necrosis in the post-treatment setting, rather than tumor below the tracer’s detection threshold. This limitation is especially relevant in the recurrent subgroup, which was small and did not include any CE−/[^18^F]FET− samples (area D). Accordingly, diagnostic performance measures in the post-treatment setting should be interpreted with caution, and the absence of systematic sampling in FLAIR+/[^18^F]FET-tissue further limits conclusions regarding the primary comparison of these imaging modalities, particularly in recurrent disease, where T2/FLAIR-positivity is a main determinant for detection of disease infiltration.

Further, sampling was opportunistic rather than grid-based and included multiple biopsies per patient, introducing potential sampling bias and within-patient clustering. Finally, spatial correspondence between preoperative imaging targets and tissue sampling may has been influenced by intraoperative brain shift, since intraoperative navigation updates were conducted based on surgeon preference rather than a predefined protocol^47^. However, an exploratory subgroup analysis addressing this dilemma showed no reduced diagnostic performance in the resection subgroup, despite this representing the more critical setting with potentially greater spatial inaccuracy than stereotactic biopsy procedures (Supplementary Table S2).

In summary, [^18^F]FET-positivity beyond contrast enhancement on MRI was highly predictive for viable tumor in IDH-wildtype glioblastoma (PPV 98.3%), indicating that CE-MRI alone may fail to detect substantial amounts of viable tumor that are captured by [^18^F]FET PET. Accordingly, CE-defined target volumes carry the risk of undertreating biologically active disease. PET-guided visualization of tumor beyond contrast enhancement may refine surgical and radiotherapy planning and should be incorporated prospectively into trials evaluating patient-tailored local treatment strategies.

## Methods

### Study participants, inclusion and exclusion criteria

This study complied with all relevant ethical regulations and was conducted in accordance with the Declaration of Helsinki. The study protocol was approved by the institutional review board of Ludwig-Maximilian-University Munich (approval nos. 17–656, 18–783, 23–0339). Written informed consent was obtained from all participants prior to imaging and surgical procedures. In this exploratory single-center, targeted sampling study conducted prospectively between October 2019 and July 2025, 147 patients with suspected newly diagnosed or recurrent diffuse glioma were enrolled. All patients underwent contrast-enhanced magnetic resonance imaging and [^18^F]FET PET prior to either stereotactic biopsy or tumor resection. Patients with histopathological diagnoses other than glioblastoma, IDH-wildtype (WHO grade 4), were excluded for this analysis. Both female and male participants were eligible for inclusion, and participant sex is reported in Table 1 and the source data. No sex-specific analyses were performed as sex-related differences were outside the scope of this diagnostic study.

### Imaging acquisition, evaluation and definition of area types

Preoperative MRI was acquired as part of routine clinical care without a study-specific standardized acquisition protocol. Accordingly, scanner hardware and sequence-specific acquisition parameters were not uniform across participants, with examinations performed at field strengths of either 1.5 or 3 Tesla. All included MRI datasets comprised T1-weighted sequences acquired before and after intravenous administration of a gadolinium-based contrast agent (around 0.1 mmol/kg body weight), as well as T2/FLAIR sequences. As part of routine clinical reporting, the contrast-enhancing component of the lesion was visually assessed and described on post-contrast T1-weighted images by two experienced neuroradiologists. Lesions were classified as contrast-enhancing if they demonstrated unequivocal signal increase relative to pre-contrast images and adjacent normal brain parenchyma. T2/FLAIR alterations were also visually assessed and documented by the same two experienced neuroradiologists. Alterations were defined as a contiguous region of hyperintense signal relative to contralateral normal-appearing white matter, excluding changes attributable to cerebrospinal fluid, vessels, or imaging artefact. [¹⁸F]FET was produced by the in-house radiopharmacy of the Department of Nuclear Medicine at LMU University Hospital Munich under good manufacturing practice conditions, subjected to routine quality control and released for clinical use. Radiochemical purity was ≥95%. Details of the radiosynthesis have been published previously^48^. Patients fasted for at least 6 h before PET imaging and received a weight-based activity of around 2.5 MBq/kg body weight (maximum 185 MBq). PET scans were acquired using Siemens Biograph PET/CT scanners (Siemens, Erlangen, Germany), employing either dynamic (0–40 min p.i.) or static (20–40 min p.i.) protocols. Patients were positioned carefully with head fixation to minimize motion artifacts while ensuring comfort. The examinations were initiated with a low-dose CT for attenuation correction. Dynamic PET data were acquired in list mode and reconstructed using the OSEM3D algorithm (4 iterations, 21 subsets) with a 5 mm Gaussian post-reconstruction filter. The reconstructed matrix size was 168 × 168 × 109, corresponding to a voxel size of 2.0 × 2.0 × 2.0 mm³. Standard corrections were applied, including attenuation, scatter, random coincidences, dead time, and radioactive decay. PET images were analyzed on a Hermes workstation (Hermes Medical Solutions, Stockholm, Sweden) using static summed images from 20 to 40 min post-injection and following established methodologies and PET RANO 1.0 criteria^44^. Accordingly, the mean activity of 4–6 adjacent, crescent-shaped regions of interest in the healthy contralateral hemisphere was measured as background activity^49^. [^18^F]FET-positivity was assessed quantitatively and defined as a standard uptake value (SUV) of 1.6× the mean background activity or higher.

The different area types of this study were determined based on the binary presence or absence of contrast enhancement on MRI and [^18^F]FET-positivity:

Area type A: CE-positive and [^18^F]FET-positive (CE+/[^18^F]FET+)

Area type B: CE-negative but [^18^F]FET-positive (CE−/[^18^F]FET+)

Area type C: CE-positive but [^18^F]FET-negative (CE+/[^18^F]FET−)

Area type D: CE-negative and [^18^F]FET-negative (CE−/[^18^F]FET−)

In addition, each area type can be supplemented with either 0 or 1, depending on whether a T2/FLAIR-alteration is absent or present (e.g., B0 = CE–, [^18^F]FET+, T2/FLAIR−).

### Tissue sampling and documentation

Tissue samples were obtained either via stereotactic biopsy or during tumor resection. All imaging datasets were preoperatively coregistered and imported into the neuronavigation system (Brainlab SE, Munich, Germany). Target regions were first described by a board-certified Nuclear Medicine physician with >15 years of experience in assessing [^18^F]FET PET images of CNS tumors (NLA), with a primary focus on area A (CE+/[^18^F]FET+) and area B (CE−/[^18^F]FET+). Final target points within these regions were then defined by board-certified neurosurgeons (SVK, VS, JCT, NT, JB, TG, SQ, FR, DK), taking into account surgical access, proximity to eloquent structures, and the planned resection corridor.

Neuronavigation was performed using the Brainlab Curve navigation system (Cranial Navigation 3.1, Cranial Navigation 4.1, and Cranial Registration 3.6 software; Brainlab SE, Munich, Germany) for both tissue sampling and tumor resection. When applying surface-based registration, the system provides a mean localization error of ≤2.0 mm and a mean trajectory angle error of ≤2°. In frame-based stereotactic procedures, distance and angular measurements have an accuracy of ±0.05 mm and ±0.05°, respectively. Intraoperative accuracy was verified in all open procedures using anatomical landmarks. To minimize the impact of intraoperative brain shift, tissue sampling for study purposes was preferentially performed prior to tumor resection. In cases involving deep-seated targets, sampling was conducted during tumor resection following careful confirmation of navigational accuracy using anatomical landmarks. Targeted intraoperative tissue sampling was guided by neuronavigation and performed under microscopic visualization using fine grasping forceps, yielding specimens with an approximate volume of 3–6 mm³.

All biopsies were obtained along predefined stereotactic trajectories and numerically encoded. Based on coregistered images, a distal target point within the target lesion and the corresponding access trajectory were planned on a stereotactic workstation. For each trajectory, the distal tip was defined as the reference level 0 ( = target point). Proximal sampling sites along the trajectory were then assigned negative integer levels in 1-mm increments, such that each millimeter step corresponded to a decrement of −1 (e.g., a biopsy taken 6 mm proximal to the trajectory’s target point was labelled −6). The corresponding level designation was recorded intraoperatively in a structured sampling log and on the individual sample containers to ensure unambiguous tracking of each specimen.

In cases of surgical resection without prior sampling, the craniotomy and cortical entry zone were planned at the discretion of the operating neurosurgeon. Registration of the neuronavigation system (Brainlab Curve, Brainlab SE, Munich, Germany) was performed using surface matching registration. Accuracy of the registration was checked against anatomical landmarks. After craniotomy and dural opening, neuronavigation was used to define sampling sites within pre-specified target areas using the navigated pointer. Tissue samples were obtained from each navigated site using standard microsurgical instruments. Each specimen was placed in a separate container, and the corresponding target ID was recorded in the sampling log and data storage of the neuronavigation system.

**Fig. 2:**
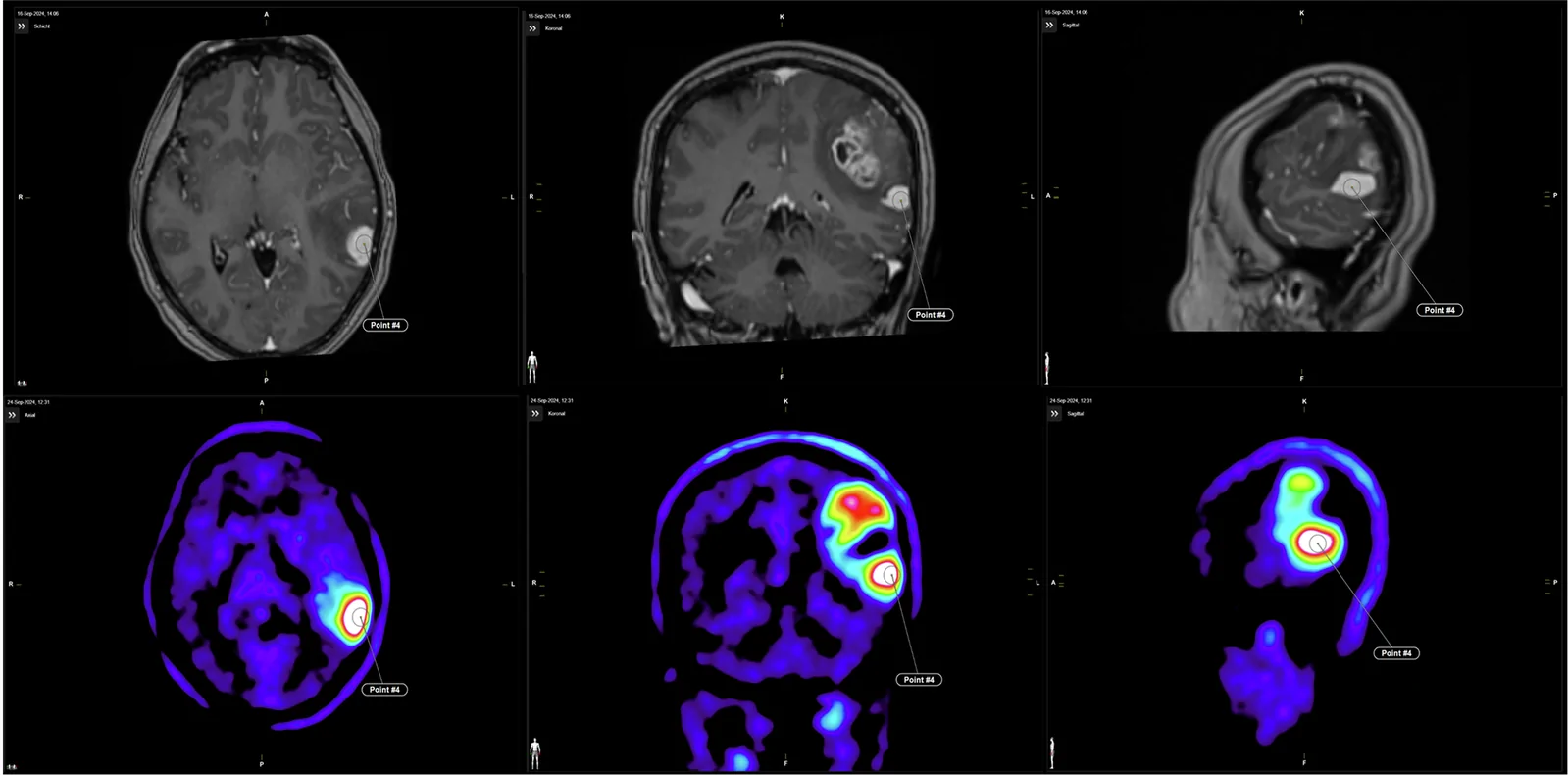
Example of precise sample coordinate documentation and integration of anatomical localization into the preoperative imaging dataset within the neuronavigation system. The sampling site is indicated by a red dot and was used to extract local imaging information from O-(2-[¹⁸F]fluoroethyl)-L-tyrosine positron emission tomography ([^18^F]FET PET) for correlation with histopathological findings. From left to right, axial, coronal, and sagittal planes are shown. Upper row: contrast-enhanced T1-weighted magnetic resonance imaging (CE-MRI); lower row: [^18^F]FET PET.

The final 3D navigation coordinates of both microsurgically and stereotactically obtained tissue samples were exported from the navigation system and burned into preoperative imaging in order to enable exact spatial correlation of imaging information and molecular findings (Fig. 2). Additionally, for each specimen, the presence or absence of contrast enhancement, T2/FLAIR-alteration and [^18^F]FET-positivity was postoperatively documented in the structured sampling log by board-certified neurosurgeons (SVK, VS, JCT, NT, JB, TG, SQ, FR, DK).

### Histopathological and molecular evaluation

All tumors were classified according to the 2021 WHO Classification of Tumors of the Central Nervous System, integrating histology and molecular markers^22^. As mentioned before, only glioblastoma, IDH-wildtype (WHO grade 4), was included in this analysis. Histopathological and molecular work-up, including formalin fixation, paraffin embedding, sectioning, and routine hematoxylin-eosin staining as well as immunohistochemistry and molecular testing, were performed by board-certified neuropathologists (VCR, PH) at the Department of Neuropathology, LMU University Hospital Munich, as part of routine neuropathological assessment.

For the purposes of this study, H&E slides of all samples were re-reviewed by a board-certified neuropathologist (MJR) at the Department of Neuropathology, University Hospital Regensburg, who visually and semiquantitatively assigned each specimen to one of three categories (0–2) based on tumor cell density and infiltration pattern (Fig. 1c), while being blinded to all clinical data. The criteria used were consistent with previously described distinctions between infiltrative margin and solid tumor components in glioblastoma^50,51^:

0 – No tumor: Cortex and/or white matter without unequivocally identifiable neoplastic glial cells on histology, with preserved cortical lamination and white-matter architecture. Apart from at most mild reactive gliosis, therapy-related changes and processing-related shrinkage artefacts, glial nuclei showed no tumor-typical pleomorphism or hyperchromasia.

1 – Infiltration zone: Moderately increased overall cell density compared to adjacent cortex/white matter, but still with a recognizable admixture of non-neoplastic elements. Entrapped cortical neurons remained clearly identifiable and were often surrounded by small clusters of atypical glial cells consistent with perineuronal satellitosis, together with scattered infiltrating tumor cells in otherwise largely preserved parenchyma. Confluent sheets of tumor, extensive necrosis, and florid microvascular proliferation typical of the tumor core were absent.

2 – Solid tumor: Densely cellular glioblastoma with near-complete effacement of normal cortical or white-matter architecture and only rare residual neurons or glial cells of non-neoplastic morphology. Tumor cells formed confluent, pleomorphic proliferations with variable mitotic activity. Microvascular proliferation and/or necrosis were common but not required for the biopsy-level definition of solid tumor, which was based on compact tumor-cell growth and replacement of normal brain parenchyma.

### Correlation of sampling location, imaging information and histopathology

For precise spatial correlation of imaging information with histopathology, all datasets were re-imported onto a Hermes workstation and analyzed using Hermes’ HERMIA Hybrid Viewer PDR version 6.1.4 (Hermes Medical Solutions, Stockholm, Sweden). Preoperative [^18^F]FET PET, contrast-enhanced T1-weighted MRI, and T2/FLAIR sequences were rigidly coregistered to the dataset containing the embedded biopsy or resection sampling coordinates (stereotactic CT or MRI). The resulting spatial alignment was carefully reviewed and, if necessary, refined based on anatomical landmarks including cortical sulci, ventricular borders, and dural reflections, analogous to previous studies using multimodal imaging-biopsy co-localization in diffuse glioma^52^.

For each tissue sample, the exact sampling coordinate was first identified on the burned-in stereotactic CT or MRI images, where the intraoperative sampling site was marked by a small cross or dot. Using the “*Triangulate”* function in Hermes’ HERMIA Hybrid Viewer, the identical location was then projected onto all coregistered imaging datasets ([^18^F]FET PET, contrast-enhanced T1-weighted MRI, and T2/FLAIR). On the corresponding PET slice, a small circular region of interest (ROI) was subsequently placed using the integrated “*Quick ROI*” tool, centered on this triangulated coordinate and confined to the presumed sampling site. The ROI was chosen to encompass only the immediate vicinity of the sampling location while minimizing partial-volume contamination from adjacent tissue or cerebrospinal fluid spaces. Within this ROI, quantitative PET parameters (SUV_mean_, SUV_max_, TBR_mean_ and TBR_max_) were extracted on the static 20–40 min [^18^F]FET PET images according to the previously defined background region and TBR calculation. For eight specimens, postoperative exporting of the precise sampling coordinates failed, and these samples were consequently excluded from analyses of quantitative PET parameters. The presence or absence of contrast enhancement, T2/FLAIR alteration, and [^18^F]FET-positivity (TBR_max_ ≥ 1.6) at each sampling site was verified on the coregistered images (SVK, RS). Findings were cross-referenced with the neuroradiologists’ visual assessment and the postoperative documentation in the structured sampling log. Each specimen was thus assigned to one of the predefined area types (A-D, with T2/FLAIR subcategories 0/1) and linked to its corresponding PET metrics and MRI characteristics. Histopathological information, including tumor cell density categories, was merged with the imaging dataset only after completion of all PET measurements and MRI assessments, such that imaging evaluation was performed blinded to the histopathological results of the corresponding tissue samples.

### Statistical analysis

All statistical analyses were performed using IBM SPSS Statistics, version 31.0 (IBM Corp., Armonk, NY, USA), and R, version 4.5.2 (R Foundation for Statistical Computing, Vienna, Austria), with the DescTools package version 0.99.60. Unless stated otherwise, tests were two-sided. All diagnostic metrics, including sensitivity, specificity, positive and negative predictive values (PPV, NPV), were calculated for [^18^F]FET PET, contrast-enhanced MRI, and T2/FLAIR as well as for each region type (A-D). Paired categorical comparisons were assessed using McNemar’s test, while unpaired comparisons of proportions were assessed with the chi-squared test (or Fisher’s exact test when expected cell counts were <5). ROC analysis was conducted to assess the diagnostic performance of TBR_max_ for distinguishing tumor-negative from tumor-positive samples. The AUC was calculated, and the data-driven cut-off was derived by maximizing the Youden index. To evaluate whether the proportion of tumor-positive biopsies deviated from chance, we used a two-sided exact binomial test against *p* = 0.5. Associations between PET-derived metrics and our semiquantitative histopathological classification were assessed using Spearman’s rank correlation and the Kruskal–Wallis test. In addition, a one-sided Jonckheere–Terpstra test was used to test for a monotonic ordered trend of increasing PET uptake across the categories no tumor, infiltration zone and solid tumor. Results were considered statistically significant at *p* < 0.05.

### Reporting summary

Further information on research design is available in the Nature Portfolio Reporting Summary linked to this article.

## Supplementary information

Supplementary Information

Reporting Summary

Transparent Peer Review file

## Source data

Source Data

## Data Availability

The de-identified patient- and sample-level data, including processed imaging-derived variables and histopathological classifications, are provided with this paper in the Source Data file. The original clinical [¹⁸F]FET PET and MRI datasets and original histopathological source material are not publicly available because they contain, or are linked to, potentially identifiable patient information and are subject to institutional, ethical and data-protection restrictions. Access to these data may be requested from the corresponding author for scientific research purposes and is subject to approval by the responsible institution and applicable data-protection requirements. Requests will be evaluated within approximately 4 weeks, and if access is approved, data will be made available for the period specified in the respective data-sharing agreement. Source data are provided with this paper.
