# Supplementary material for: [18F]FET PET enables histopathology-validated detection of non-contrast-enhancing glioblastoma
Source: Nat Commun. 2026 Nov 6;17:11498. doi: 10.1038/s41467-026-78980-1 (PMC13743775; doi:10.1038/s41467-026-78980-1)
Supplement: Supplementary file 1 [file 41467_2026_78980_MOESM1_ESM.pdf]

Supplementary Information

	No tumor (*n*, %)	Infiltration zone (*n*, %)	Solid tumor (*n*, %)	Overall (*n*, %)	
**A0**	0 (0.0)	6 (4.3)	22 (12.1)	28 (7.4)	139 (36.9)
**A1**	1 (1.9)	31 (22.0)	79 (43.4)	111 (29.4)	
**B0**	2 (3.7)	14 (9.9)	2 (1.1)	18 (4.8)	153 (40.6)
**B1**	1 (1.9)	56 (39.7)	78 (42.9)	135 (35.8)	
**C0**	1 (1.9)	7 (5.0)	0 (0.0)	8 (2.1)	19 (5.0)
**C1**	6 (11.1)	5 (3.5)	0 (0.0)	11 (2.9)	
**D0**	14 (25.9)	6 (4.3)	0 (0.0)	20 (5.3)	66 (17.5)
**D1**	29 (53.7)	16 (11.3)	1 (0.5)	46 (12.2)	
**Overall**	54 (100.0)	141 (100.0)	182 (100.0)	377 (100.0)	

Tab. S1A: Tissue sample distribution in initial diagnosis. Exact distribution across area types A-D, including binary coding for the absence (0) or presence (1) of T2/FLAIR alteration, divided according to histological strata and overall, in the initial diagnosis cohort.

	No tumor (*n*, %)	Infiltration zone (*n*, %)	Solid tumor (*n*, %)	Overall (*n*, %)	
**A0**	4 (44.4)	0 (0.0)	0 (0.0)	4 (4.2)	71 (74.7)
**A1**	3 (33.3)	34 (66.7)	30 (85.7)	67 (70.5)	
**B0**	0 (0.0)	3 (5.9)	0 (0.0)	3 (3.2)	21 (22.1)
**B1**	0 (0.0)	14 (27.5)	4 (11.4)	18 (18.9)	
**C0**	0 (0.0)	0 (0.0)	0 (0.0)	0 (0.0)	3 (3.2)
**C1**	2 (22.2)	0 (0.0)	1 (2.9)	3 (3.2)	
**D0**	0 (0.0)	0 (0.0)	0 (0.0)	0 (0.0)	0 (0.0)
**D1**	0 (0.0)	0 (0.0)	0 (0.0)	0 (0.0)	
**Overall**	9 (100.0)	51 (100.0)	35 (100.0)	95 (100.0)	

Tab. S1B: Tissue sample distribution in recurrence. Exact distribution across area types A-D, including binary coding for the absence (0) or presence (1) of T2/FLAIR alteration, divided according to histological strata and overall, in the recurrence cohort.

Imaging modality	Diagnostic metric	Stereotactic biopsy	Resection	*p*-value
**T2/FLAIR**	Sensitivity	85.1%	86.0%	0.877
	Specificity	33.3%	33.3%	1.000
	PPV	87.5%	94.2%	0.064
	NPV	29.0%	15.8%	0.372
**CE-MRI**	Sensitivity	47.5%	65.8%	0.001
	Specificity	77.8%	44.4%	0.052
	PPV	92.1%	93.8%	0.794
	NPV	21.3%	9.3%	0.087
**[^{18}F]FET PET**	Sensitivity	89.5%	95.6%	0.053
	Specificity	81.5%	88.9%	1.000
	PPV	96.4%	99.1%	0.190
	NPV	58.7%	61.5%	1.000

Supplementary Table S2: Diagnostic performance by sampling method. Sensitivity, specificity, positive predictive value (PPV), and negative predictive value (NPV) are reported as percentages for detecting histology-verified tumor, stratified by sampling method (stereotactic biopsy vs. surgical resection) and imaging modality ([^{18}F]FET PET, CE-MRI, and T2/FLAIR). P values were calculated using two-sided Fisher’s exact tests and no adjustment for multiple comparisons was applied.
