# Supplementary material for: [18F]FET PET enables histopathology-validated detection of non-contrast-enhancing glioblastoma
Source: Nat Commun. 2026 Nov 6;17:11498. doi: 10.1038/s41467-026-78980-1 (PMC13743775; doi:10.1038/s41467-026-78980-1)
Supplement: Supplementary file 2 [file 41467_2026_78980_MOESM2_ESM.pdf]

nature portfolio

Corresponding author(s): Prof. Dr. Nathalie Lisa Albert

Last updated by author(s): Sep 29, 2026

Reporting Summary

Nature Portfolio wishes to improve the reproducibility of the work that we publish. This form provides structure for consistency and transparency in reporting. For further information on Nature Portfolio policies, see our Editorial Policies and the Editorial Policy Checklist.

Statistics

For all statistical analyses, confirm that the following items are present in the figure legend, table legend, main text, or Methods section.

n/a	Confirmed	
☐	☒	The exact sample size (n) for each experimental group/condition, given as a discrete number and unit of measurement
☐	☒	A statement on whether measurements were taken from distinct samples or whether the same sample was measured repeatedly
☐	☒	The statistical test(s) used AND whether they are one- or two-sided<br>*Only common tests should be described solely by name; describe more complex techniques in the Methods section.*
☒	☐	A description of all covariates tested
☐	☒	A description of any assumptions or corrections, such as tests of normality and adjustment for multiple comparisons
☐	☒	A full description of the statistical parameters including central tendency (e.g. means) or other basic estimates (e.g. regression coefficient) AND variation (e.g. standard deviation) or associated estimates of uncertainty (e.g. confidence intervals)
☐	☒	For null hypothesis testing, the test statistic (e.g. F, t, r) with confidence intervals, effect sizes, degrees of freedom and P value noted<br>*Give P values as exact values whenever suitable.*
☒	☐	For Bayesian analysis, information on the choice of priors and Markov chain Monte Carlo settings
☐	☒	For hierarchical and complex designs, identification of the appropriate level for tests and full reporting of outcomes
☐	☒	Estimates of effect sizes (e.g. Cohen's d, Pearson's r), indicating how they were calculated

Our web collection on statistics for biologists contains articles on many of the points above.

Software and code

Policy information about availability of computer code

Data collection	Image-guided tissue sampling, stereotactic trajectory planning, neuronavigation, and spatial documentation of the sampling coordinates were performed using the Brainlab Curve navigation system with Cranial Navigation versions 3.1 and 4.1 and Cranial Registration version 3.6 (Brainlab SE, Munich, Germany). No custom software or code was used for data collection.
Data analysis	Image evaluation, multimodal rigid image coregistration, triangulation of tissue-sampling coordinates, placement of regions of interest, and extraction of quantitative SUV and TBR parameters were performed using HERMIA Hybrid Viewer PDR version 6.1.4 on a Hermes workstation (Hermes Medical Solutions, Stockholm, Sweden). Statistical analyses were performed using IBM SPSS Statistics version 31.0 (IBM Corp., Armonk, NY, USA) and R version 4.5.2 (R Foundation for Statistical Computing, Vienna, Austria), with the DescTools package version 0.99.60. No custom software or code was used for data collection.

For manuscripts utilizing custom algorithms or software that are central to the research but not yet described in published literature, software must be made available to editors and reviewers. We strongly encourage code deposition in a community repository (e.g. GitHub). See the Nature Portfolio guidelines for submitting code & software for further information.

Data

Policy information about availability of data

All manuscripts must include a data availability statement. This statement should provide the following information, where applicable:

- Accession codes, unique identifiers, or web links for publicly available datasets
- A description of any restrictions on data availability
- For clinical datasets or third party data, please ensure that the statement adheres to our policy

The deidentified patient- and sample-level data including processed imaging-derived variables and histopathological classifications, are provided with this paper in the Source Data file. The original clinical [^{18}F]FET PET and MRI datasets and original histopathological source material are not publicly available because they contain, or are linked to, potentially identifiable patient information and are subject to institutional, ethical and data-protection restrictions. Access to these data may be requested from the corresponding author for scientific research purposes and is subject to approval by the responsible institution and applicable data-protection requirements. Requests will be evaluated within approximately 4 weeks and if access is approved, data will be made available for the period specified in the respective data-sharing agreement. Source data are provided with this paper.

Research involving human participants, their data, or biological material

Policy information about studies with human participants or human data. See also policy information about sex, gender (identity/presentation), and sexual orientation and race, ethnicity and racism.

Reporting on sex and gender	Sex was recorded as female or male and is reported in Table 1 and the source data. The study included 26 female and 35 male participants. Sex was obtained from clinical records. Gender identity was not collected. Sex-specific analyses were not performed because the primary analyses were conducted at the tissue-sample level and the study was not designed or powered to assess sex-related differences in imaging performance.
Reporting on race, ethnicity, or other socially relevant groupings	Race, ethnicity, and other socially constructed or socially relevant grouping variables were not collected or analyzed in this study. These variables were not used as proxies for socioeconomic status or other clinical characteristics. Accordingly, no race- or ethnicity-based analyses or confounder adjustments were performed.
Population characteristics	The study included 61 adult patients with histopathologically confirmed glioblastoma, IDH-wildtype, WHO grade 4, classified according to the 2021 WHO Classification of Tumors of the Central Nervous System. Median age was 59 years. 26 participants were female and 35 were male. Forty-five patients had newly diagnosed disease and 16 had recurrent disease. Twenty-six patients underwent stereotactic biopsy and 35 underwent tumor resection. All patients underwent preoperative contrast-enhanced MRI and [18F]FET PET. Detailed population characteristics are reported in Table 1 and the Source date file.
Recruitment	Patients were prospectively enrolled at a single academic center between October 2019 and July 2025. Eligible participants had suspected newly diagnosed or recurrent diffuse glioma and underwent contrast-enhanced MRI and [18F]FET PET before stereotactic biopsy or tumor resection. Of 147 prospectively enrolled patients, those with histopathological diagnoses other than glioblastoma, IDH-wildtype, WHO grade 4, were excluded from the present analysis, resulting in a final cohort of 61 patients.
Ethics oversight	The study was approved by the institutional review board of Ludwig-Maximilians-Universität Munich, Germany, under approval numbers 17-656, 18-783, and 23-0339. The study was conducted in accordance with the Declaration of Helsinki. Written informed consent was obtained from all participants before imaging and surgical procedures.

Note that full information on the approval of the study protocol must also be provided in the manuscript.

Field-specific reporting

Please select the one below that is the best fit for your research. If you are not sure, read the appropriate sections before making your selection.

[x] Life sciences [ ] Behavioural & social sciences [ ] Ecological, evolutionary & environmental sciences

For a reference copy of the document with all sections, see nature.com/documents/nr-reporting-summary-flat.pdf

Life sciences study design

All studies must disclose on these points even when the disclosure is negative.

Sample size	No statistical method was used to predetermine the sample size. As it is a single-center targeted-sampling study, the sample size was determined by the number of patients meeting the study eligibility criteria during the recruitment period and the number of spatially matched, histopathologically evaluable tissue samples obtained from them. The resulting cohort of 61 patients and 472 tissue samples included more patients and histopathologically evaluated samples than two previous biopsy-validated FET PET/MRI studies (Harat et al., 2023 [10]; Mauler et al., 2024[20]).
Data exclusions	Of 147 prospectively enrolled patients with suspected newly diagnosed or recurrent diffuse glioma, patients with histopathological diagnoses other than glioblastoma, IDH-wildtype, WHO grade 4, were excluded from the present analysis. For 8 of the 472 tissue samples, quantitative

PET parameters could not be obtained because postoperative export of the precise sampling coordinates failed. These samples were excluded only from analyses of quantitative PET parameters but were retained in analyses based on categorical imaging and histopathological information. No additional post hoc data exclusions were made.

Replication

Independent replication in a separate patient cohort was not performed. Each tissue specimen represented a distinct biological sample, and multiple spatially distinct specimens could be obtained from the same patient. The robustness of the main findings was assessed in subgroup analyses according to disease stage and sampling method, with concordant results in newly diagnosed vs. recurrent disease and in stereotactic biopsy vs. resection specimens.

Randomization

Randomization was not applicable because this was a non-interventional diagnostic-accuracy study without allocation to treatment or experimental groups. Tissue-sampling locations were not randomly selected but were targeted on the basis of the imaging-defined regions, surgical accessibility, proximity to eloquent structures, and the planned surgical trajectory or resection corridor.

Blinding

Histopathological re-review and semiquantitative classification of all tissue samples were performed by a board-certified neuropathologist whowasblinded to all clinical data. Imaging evaluation and quantitative PET measurements were completed before the histopathological information was merged with the imaging dataset. Therefore, imaging assessment was performed blinded to the corresponding histopathological results. Intraoperative target selection and tissue sampling were not blinded because these procedures were intentionally guided by the preoperative PET and MRI findings and by clinical and surgical considerations.

Reporting for specific materials, systems and methods

We require information from authors about some types of materials, experimental systems and methods used in many studies. Here, indicate whether each material, system or method listed is relevant to your study. If you are not sure if a list item applies to your research, read the appropriate section before selecting a response.

Materials & experimental systems

n/a	Involved in the study	
x		Antibodies
x		Eukaryotic cell lines
x		Palaeontology and archaeology
x		Animals and other organisms
	x	Clinical data
x		Dual use research of concern
x		Plants

Methods

n/a	Involved in the study	
x		ChIP-seq
x		Flow cytometry
	x	MRI-based neuroimaging

Clinical data

Policy information about clinical studies

All manuscripts should comply with the ICMJE guidelines for publication of clinical research and a completed CONSORT checklist must be included with all submissions.

Clinical trial registration

Not applicable. This was a targeted-sampling study without assignment of participants to therapeutic or diagnostic interventions and was therefore not registered as a clinical trial.

Study protocol

The full study protocol is not publicly available. The study design, eligibility criteria, imaging procedures, tissue-sampling workflow, histopathological assessment and statistical methods are described in detail in the Methods section of the manuscript. The study was conducted according to protocols approved by the institutional review board of Ludwig-Maximilians-Universität Munich.

Data collection

Data were collected at LMU University Hospital Munich, Germany, between October 2019 and July 2025. Patients were screened for eligibility during routine clinical care and all patients meeting the inclusion criteria were invited to participate and asked to provide informed consent. Participation was voluntary and did not involve allocation to different treatment groups. As participation was voluntary, some self-selection bias cannot be excluded. In addition, inclusion required that both imaging examinations could be completed before clinically indicated surgical procedure, which may have limited the inclusion of patients requiring more urgent intervention. These factors may limit the generalizability of the findings to such patients.

Outcomes

This was an exploratory study without a formally registered primary endpoint. The principal outcome was the specimen-level diagnostic performance of [18F]FET PET compared with contrast-enhanced MRI for detecting histopathologically confirmed tumor, assessed by sensitivity, specificity, positive predictive value and negative predictive value using spatially matched histopathology as the reference standard. Secondary and exploratory outcomes included diagnostic performance across imaging-defined areas A–D and in comparison with T2/FLAIR, quantitative SUV and TBR analyses, ROC-based evaluation of the TBRmax threshold, associations between PET uptake and semiquantitative tumor burden, and subgroup analyses by disease stage and sampling method.

Plants

Seed stocks | Not applicable.

Novel plant genotypes | Not applicable.

Authentication | Not applicable.

Magnetic resonance imaging

Experimental design

Design type | Not applicable to task- or resting-state paradigms. This was an observational structural neuroimaging study using preoperative clinical MRI acquired as part of routine care.

Design specifications | No event-related, block or trial design was used. For each participant, a preoperative clinical MRI dataset comprising pre- and post-contrast T1-weighted imaging and T2/FLAIR imaging was evaluated.

Behavioral performance measures | Not applicable. No behavioral task or behavioral performance measures were collected.

Acquisition

Imaging type(s) | Structural MRI, including T1-weighted imaging before and after administration of a gadolinium-based contrast agent and T2/FLAIR imaging. Functional, diffusion and perfusion MRI were not analyzed.

Field strength | MRI examinations were acquired as part of routine clinical care without a study-specific standardized acquisition protocol. Consequently, scanner hardware and acquisition parameters were not uniform across participants, with examinations performed at field strengths of either 1.5 or 3 Tesla.

Sequence & imaging parameters | MRI acquisition parameters were not standardized because examinations were obtained using routine clinical protocols. All analyzed datasets included T1-weighted imaging before and after administration of a gadolinium-based contrast agent at approximately 0.1 mmol/kg body weight, as well as T2/FLAIR imaging. Sequence-specific parameters, including TR, TE, flip angle, field of view, matrix size, slice thickness and orientation, varied according to scanner and clinical protocol and were not used as study variables.

Area of acquisition | Whole-brain clinical MRI datasets covering the intracranial compartment, tumor region and surrounding brain tissue were analyzed.

Diffusion MRI ☐ Used ☒ Not used

Preprocessing

Preprocessing software | MRI datasets were re-imported and processed using HERMIA Hybrid Viewer PDR version 6.1.4 on a Hermes workstation (Hermes Medical Solutions, Stockholm, Sweden). Preoperative contrast-enhanced T1-weighted and T2/FLAIR images were rigidly coregistered within each participant to the stereotactic CT or MRI dataset containing the embedded tissue-sampling coordinates. Spatial alignment was visually reviewed and, where necessary, manually refined.

Normalization | No spatial normalization to a common anatomical or standard template was performed. All analyses were conducted in the participant-specific native imaging space after rigid within-subject coregistration.

Normalization template | Not applicable. No normalization template was used.

Noise and artifact removal | No dedicated MRI denoising or physiological-noise correction procedure was applied. Image quality and coregistration accuracy were visually reviewed, and apparent imaging artifacts were considered during image interpretation.

Volume censoring | Not applicable. No functional MRI or MRI time-series analysis was performed, and no volumes were censored.

Statistical modeling & inference

Model type and settings | Not applicable. No voxel-wise or cluster-based neuroimaging model was applied. MRI findings were assessed categorically at spatially matched tissue-sampling locations, and statistical analyses were performed at the specimen level.

Effect(s) tested | Not applicable to task- or stimulus-related neuroimaging effects. The analyses compared imaging findings from [18F]FET PET, contrast-enhanced MRI and T2/FLAIR with histopathologically confirmed tumor status at spatially matched tissue-sampling

locations.

Specify type of analysis: ☐ Whole brain ☒ ROI-based ☐ Both

Anatomical location(s)

Anatomical locations were defined individually using the neuronavigation-derived tissue-sampling coordinates rather than automated anatomical labeling or probabilistic atlases. The exact sampling coordinates embedded in the stereotactic CT or MRI dataset were projected onto the rigidly coregistered contrast-enhanced T1-weighted, T2/FLAIR and [18F]FET PET images using the "Triangulate" function in HERMIA Hybrid Viewer. Registration was visually verified and, if necessary, refined using anatomical landmarks. Quantitative PET ROIs were centered on the corresponding sampling coordinates, and MRI features were assessed at the same locations in participant-specific native space. No normalization to a standard template was performed.

Statistic type for inference

(See Eklund et al. 2016)

Not applicable. No voxel-wise or cluster-wise neuroimaging inference was performed.

Correction

Not applicable. No voxel-wise multiple-comparison correction, such as FWE or FDR correction, was required because no whole-brain or cluster-based neuroimaging analysis was performed.

Models & analysis

n/a	Involved in the study
☒	☐ Functional and/or effective connectivity
☒	☐ Graph analysis
☒	☐ Multivariate modeling or predictive analysis
