# Supplementary material for: [18F]FET PET enables histopathology-validated detection of non-contrast-enhancing glioblastoma
Source: Nat Commun. 2026 Nov 6;17:11498. doi: 10.1038/s41467-026-78980-1 (PMC13743775; doi:10.1038/s41467-026-78980-1)
Supplement: Supplementary file 3 [file 41467_2026_78980_MOESM3_ESM.pdf]

nature portfolio

Peer Review File

[^{18}F]FET PET enables histopathology-validated detection of non-contrast-enhancing glioblastoma

Corresponding Author: Professor Nathalie Albert

This file contains all reviewer reports in order by version, followed by all author rebuttals in order by version.

Version 1:

Reviewer comments:

Reviewer #1

(Remarks to the Author)
This manuscript presents a prospective biopsy-validated study evaluating the ability of ^{18}F-FET PET to detect viable tumor tissue beyond contrast-enhancing MRI in IDH-wildtype glioblastoma. The authors analyze 61 patients and 472 spatially matched tissue samples, constituting the largest histopathology-correlated datasets in this field.

The results demonstrate the superior sensitivity of FET PET compared with CE-MRI for tumor detection. Using a TBR threshold ≥1.6, FET PET achieved a sensitivity of 91.2%, markedly higher than 55.7% for CE-MRI, with comparable specificity (82.5% vs 73.0%). Notably, in non-contrast-enhancing regions (CE−/FET+), the positive predictive value reached 98.3%, indicating that metabolically active tumor frequently extends beyond the MRI contrast boundary. These findings provide robust histopathological evidence that amino-acid PET reveals tumor burden not captured by contrast-enhanced MRI.

The study is very well designed and executed. The large number of spatially correlated biopsies, the homogeneous IDH-wildtype cohort, and the careful multimodal co-registration between imaging and histopathology make this dataset particularly valuable. The manuscript is clearly written and the conclusions are well supported by the data.

I only have a few minor suggestions that could further strengthen the manuscript:
- Dynamic acquisitions were performed in a subset of patients, yet the analysis focuses exclusively on static parameters. A brief discussion of the potential contribution of dynamic FET metrics for characterizing tumor infiltration would be interesting.

- Table 1 indicates that five patients did not show contrast enhancement. It would be helpful to clarify whether all CE-negative tumors were FET-positive, and whether these cases showed specific molecular characteristics.

- Figures 1 and 2 could benefit from improved slice orientation.

Overall, this is a high-quality and clinically relevant study providing strong histopathological validation of the added value of FET PET in glioblastoma.

Reviewer #2

(Remarks to the Author)
This manuscript prospectively enrolled patients on a study where histopathological assessment was spatially linked to FET-PET and MRI scans in WHO grade 4 glioblastomas (w/IDH wild type). They examined 472 specimens from 61 patients where each specimen was spatially linked with corresponding pre-operative FET-PET and MRI scans with each being labeled as CE+/FET+ (A), CE-/FET+ (B), CE+/FET- (C) and CE-/FET- (D) with further classification without (0) or with (1) FLAIR signal. FET was considered + if value was >1.6-fold the mean background activity. They found that FET-PET showed greater sensitivity, specificity, PPV and NPV for detecting presence of tumor than CE- or FLAIR-MRI. I feel the data is quite strong for supporting FET-PET as an imaging modality that can supplement standard MRI for identifying regions of GBM tumor involvement not picked up by CE-MRI. Overall, this is one of the largest study where an advanced imaging modality is

assessed/correlated with histopathologic evaluation. I do feel that this manuscript can be further improved if the following issues are addressed:

1) The presented histologic evaluations are really mainly qualitiative to, at most, semi-quantitative in nature. Presenting more data with quantitative histologic assessments (e.g. actual tumor cell density) correlated with the FET-PET scan to see how well the strength of the PET signal correlates with the actual tumor cell density would add significantly greater strength to the findings.
2) Would have liked to see data that presents the distance of the evaluated histologic specimens from the closest contrast enhancing disease. Because RT is generally targeted to contrast enhancing disease with some level of margin, demonstrating that FET-PET also performs well further away from contrast enhancing disease will increase confidence that this study will identify infiltrative disease more distant from that identified by CE-MRI.
3) Another assessment that can get at this above question would be to see how well FET-PET performed in the 5 pts where there was no contrast enhancing disease present. If performance was similar to the group as a whole, that would provide greater assurance that FET-PET will perform similarly even when it is more distant from contrast enhancing disease.
4) (minor) Would like to see another table showing sensitivity/specificity/PPV/NPV of + imaging tests (CE+ and/or FET+, or pooling of groups A-C) vs – imaging tests (CE- and FET-, or group D alone) to see performance when using these as complementary imaging tests. I know this can be derived from the data that is presented but would lay this out more easily for the reader.

Reviewer #3

(Remarks to the Author)
Study summary:
This is a well-executed study addressing the challenging management paradigm of glioblastoma. The core finding - that [18F]FET PET detects viable tumor beyond the contrast-enhancing margin with very high positive predictive value - is supported by the largest histopathologically validated dataset in this context to date. The study is methodologically rigorous with a homogenous and well defined cohort (IDH-wildtype GBM only, classified by 2021 WHO criteria).
A major limitation to the overall relevance of the study is the clinical implications of FET PET positivity in treatment paradigm of GBM noting that recent results from the GLIAA trial did not demonstrate outcome advantage in FET PET vs CE MRI in reirradiation of recurrent GBM.
Major Comments

Spatial misregistration / brain shift
More information required on coregistration between mri and intraop navigation system. What is margin of error . How large are sample sizes ? The acknowledgment of intraoperative brain shift as an inherent limitation is insufficient without an attempt at estimating the spatial error it introduces, since the scientific value of this study rests entirely on the accuracy between preoperative imaging targets and actual tissue sampling locations. This matters most at the boundary between area types, where a 2–3 mm displacement could reclassify a sample area. The authors should report the navigation system's stated targeting accuracy, document any intraoperative accuracy checks performed, and present a sensitivity analysis restricted to stereotactic biopsy cases where spatial accuracy is highest / easier to maintain and open resection cases. Did the open resection cases start with stereotactic biopsy which was then converted to resection ?
Reproducibility of methodology
Further details regarding methodology is needed to make this a reproducible study. The authors did not specify the size of each biopsy sample acquired.
While it is apparent that multiple samples were taken from each patient, there is no detailed explanation. Did one patient have 10 samples while another had 2? Please show this as part of table 1.
What software was used for co-registration? What software version number etc.

Clarification on definition of solid tumor - histopathology

What do the authors mean by “in most cases” there is microvascular proliferation and/or necrosis. What were the exact histopathologic definition? What about mitotic figures/high mitotic rate (how many per high power field)? Are there cases included that did not have these features? If so, then are these defined based on molecular features?
“Tumor cells formed confluent, pleomorphic proliferations with frequent mitoses and, in most cases, microvascular proliferation and/or necrosis.”

FLAIR +signal as a segmentation

While the authors subdivided areas A-D by FLAIR signal, they did not evaluate FLAIR+ as a primary comparison to FET positive. In addition to contrast enhancement, FLAIR+ signal change is also used as a primary driver to evaluate for disease infiltration particularly among patients with recurrence. Consider adding this to the discussion section.
Sampling bias and under-sampling of FET-negative cases (Area C and D)
Area C contains only 22 samples, yielding a PPV of 59.1%. This under-sampling reflects the study's deliberate focus on FET-positive regions, but it leaves unresolved a clinically important question: whether PET negativity in contrast-enhancing tissue reliably identifies treatment effect, radiation necrosis, or non-tumoral blood-brain barrier disruption, particularly in the post-treatment setting. The authors should report confidence intervals in table 5, and address this shortcoming in the discussion section.
The recurrence subgroup is underpowered and Area D within recurrence is not obtained
The recurrence cohort of 17 patients and 95 samples is underpowered. Additionally, the absence of any Area D biopsies in

this group means the B vs D or A vs D comparisons where distinguishing viable tumors from treatment-related change is the central diagnostic question cannot be performed at all. The reported PPV values for Areas A and B in recurrent disease are therefore derived from a highly selected, PET-positive sample that cannot address whether FET-negative, non-enhancing tissue in this context is truly tumor-free or simply below the tracer's detection threshold after treatment-induced microstructural changes.
Table 2 is confusing to have both initial diagnosis and recurrent cases (in particular area D cohort - as it does not include any recurrent cases). Consider splitting initial and recurrent cases as the clinical question for recurrence often hinges on FLAIR changes which is not as important for initial diagnosis.
Please explain results of table 6: under recurrence group - Area A specificity = 22.2 vs Area B specificity = 100.0

Reviewer #4

(Remarks to the Author)
I co-reviewed this manuscript with one of the reviewers who provided the listed reports. This is part of the Nature Communications initiative to facilitate training in peer review and to provide appropriate recognition for Early Career Researchers who co-review manuscripts.

Version 2:

Reviewer comments:

Reviewer #1

(Remarks to the Author)
The authors have adequately addressed all the concerns raised in my previous review. I have no further comments and consider the revised manuscript suitable for publication in its current form.

Reviewer #2

(Remarks to the Author)
This manuscript prospectively enrolled patients on a study where histopathological assessment was spatially linked to FET-PET and MRI scans in WHO grade 4 glioblastomas (w/IDH wild type). They examined 472 specimens from 61 patients where each specimen was spatially linked with corresponding pre-operative FET-PET and MRI scans with each being labeled as CE+/FET+ (A), CE-/FET+ (B), CE+/FET- (C) and CE-/FET- (D) with further classification without (0) or with (1) FLAIR signal. FET was considered + if value was >1.6-fold the mean background activity. They found that FET-PET showed greater sensitivity, specificity, PPV and NPV for detecting presence of tumor than CE- or FLAIR-MRI.

I had several critiques in my initial review that the authors thoughtfully addressed. I do not have additional suggestions at this time.

Reviewer #3

(Remarks to the Author)
I thank the authors for their thorough and thoughtful revision of the manuscript. The rebuttal letter adequately addresses each of the concerns raised in the initial review. The additional analyses/clarifications strengthen the scientific rigor and clarity of the manuscript, and the revised text improves the overall readability and impact of the work. I have no further concerns and believe the manuscript is suitable for publication in its current form.

Reviewer #4

(Remarks to the Author)
I co-reviewed this manuscript with one of the reviewers who provided the listed reports. This is part of the Nature Communications initiative to facilitate training in peer review and to provide appropriate recognition for Early Career Researchers who co-review manuscripts.

Open Access This Peer Review File is licensed under a Creative Commons Attribution 4.0 International License, which permits use, sharing, adaptation, distribution and reproduction in any medium or format, as long as you give appropriate credit to the original author(s) and the source, provide a link to the Creative Commons license, and indicate if changes were made.
In cases where reviewers are anonymous, credit should be given to 'Anonymous Referee' and the source.
The images or other third party material in this Peer Review File are included in the article's Creative Commons license, unless indicated otherwise in a credit line to the material. If material is not included in the article's Creative Commons license and your intended use is not permitted by statutory regulation or exceeds the permitted use, you will need to obtain permission directly from the copyright holder.
To view a copy of this license, visit https://creativecommons.org/licenses/by/4.0/

REVIEWER COMMENTS

Reviewer #1 (Remarks to the Author):

This manuscript presents a prospective biopsy-validated study evaluating the ability of ^{18}F-FET PET to detect viable tumor tissue beyond contrast-enhancing MRI in IDH-wildtype glioblastoma. The authors analyze 61 patients and 472 spatially matched tissue samples, constituting the largest histopathology-correlated datasets in this field.

The results demonstrate the superior sensitivity of FET PET compared with CE-MRI for tumor detection. Using a TBR threshold ≥1.6, FET PET achieved a sensitivity of 91.2%, markedly higher than 55.7% for CE-MRI, with comparable specificity (82.5% vs 73.0%). Notably, in non-contrast-enhancing regions (CE−/FET+), the positive predictive value reached 98.3%, indicating that metabolically active tumor frequently extends beyond the MRI contrast boundary. These findings provide robust histopathological evidence that amino-acid PET reveals tumor burden not captured by contrast-enhanced MRI.

The study is very well designed and executed. The large number of spatially correlated biopsies, the homogeneous IDH-wildtype cohort, and the careful multimodal co-registration between imaging and histopathology make this dataset particularly valuable. The manuscript is clearly written and the conclusions are well supported by the data.

I only have a few minor suggestions that could further strengthen the manuscript:

- Dynamic acquisitions were performed in a subset of patients, yet the analysis focuses exclusively on static parameters. A brief discussion of the potential contribution of dynamic FET metrics for characterizing tumor infiltration would be interesting.

We thank the reviewer for this valuable comment. We agree that dynamic [^{18}F]FET PET may provide complementary information beyond static uptake measures. However, dynamic acquisitions were available only in a subset of patients, whereas our biopsy-controlled analysis was designed to use a uniform static threshold approach across the full cohort. We therefore focused on static parameters to ensure methodological consistency. We have now added a short statement to the Discussion acknowledging the potential role of dynamic FET metrics.

- Table 1 indicates that five patients did not show contrast enhancement. It would be helpful to clarify whether all CE-negative tumors were FET-positive, and whether these cases showed specific molecular characteristics.

We thank the Reviewer for this helpful suggestion. We have now added an exploratory subgroup analysis of the five patients without contrast enhancement on MRI to the revised Results section. All five CE-negative tumors were [^{18F}]FET-positive at the patient level. We also included a brief descriptive summary of their molecular characteristics. No obvious molecular pattern emerged: MGMT promoter methylation was present in four cases, TERT mutation in three, LOH 1p/19q was absent in all cases, and CDKN2A/B alteration was detected in one case, while four cases lacked CDKN2A/B data.

- Figures 1 and 2 could benefit from improved slice orientation.

We thank the reviewer for this valuable suggestion. We have revised Figures 1 and 2 to improve slice orientation, anatomical alignment, and overall visual comparability. For Figure 1, the images were

reoriented in the axial, coronal and sagittal planes and subsequently co-registered again to obtain the closest possible correspondence of the displayed slices across modalities. Although minor residual differences might not be entirely eliminated due to differences in slice thickness and acquisition geometry between MR sequences and imaging modalities, the revised figure now aims to provide improved anatomical matching and clearer cross-modality comparability. For Figure 2 the displayed slices were adjusted to better center the images and to avoid, as far as possible, cutting off relevant anatomical or imaging information. We also removed the crosshair to improve clarity and readability of the figure.

Overall, this is a high-quality and clinically relevant study providing strong histopathological validation of the added value of FET PET in glioblastoma.

Reviewer #2 (Remarks to the Author):

This manuscript prospectively enrolled patients on a study where histopathological assessment was spatially linked to FET-PET and MRI scans in WHO grade 4 glioblastomas (w/IDH wild type). They examined 472 specimens from 61 patients where each specimen was spatially linked with corresponding pre-operative FET-PET and MRI scans with each being labeled as CE+/FET+ (A), CE-/FET+ (B), CE+/FET- (C) and CE-/FET- (D) with further classification without (0) or with (1) FLAIR signal. FET was considered + if value was >1.6-fold the mean background activity. They found that FET-PET showed greater sensitivity, specificity, PPV and NPV for detecting presence of tumor than CE- or FLAIR-MRI. I feel the data is quite strong for supporting FET-PET as an imaging modality that can supplement standard MRI for identifying regions of GBM tumor involvement not picked up by CE-MRI. Overall, this is one of the largest study where an advanced imaging modality is assessed/correlated with histopathologic evaluation. I do feel that this manuscript can be further improved if the following issues are addressed:

1) The presented histologic evaluations are really mainly qualitiative to, at most, semi-quantitative in nature. Presenting more data with quantitative histologic assessments (e.g. actual tumor cell density) correlated with the FET-PET scan to see how well the strength of the PET signal correlates with the actual tumor cell density would add significantly greater strength to the findings.

We thank the reviewer for this important comment and understand that fully quantitative histopathological measures, like absolute tumor cell density, would be desirable. However, such “precise” data are not available in our cohort, as they may be confounded in diffusely infiltrating glioma tissue by admixture of neoplastic cells, reactive astroglia, and immune/microglial cells. Although IDH1-R132H immunohistochemistry can support the distinction between neoplastic and reactive astroglial cells in IDH-mutant gliomas, it is not applicable as a tumor-cell-specific marker in our IDH-wildtype glioblastoma cohort. Indeed, there is no single tumor-cell specific marker available in IDH-wildtype glioblastoma that allows for a reliable distinction of neoplastic from reactive astroglial cells. Nevertheless, as we agree that an analysis correlating PET signal intensity with histopathological tumor burden would strengthen the findings, we addressed this point as rigorously as possible by performing additional analyses using our three-tiered semiquantitative histopathological classification (0 = no tumor, 1 = infiltration zone, 2 = solid tumor). This classification reflects increasing tumor burden but avoids being “over-/pseudoexact”.

We have incorporated these additional analyses into the manuscript. In the Methods section, we now specify that “Associations between PET-derived metrics and our semiquantitative histopathological classification were assessed using Spearman’s rank correlation and the Kruskal–Wallis test. In addition, a one-sided Jonckheere-Terpstra test was used to test for a monotonic ordered trend of increasing PET uptake across the categories no tumor, infiltration zone and solid tumor.” In the Results, under *Robustness of [^{18}F]FET PET across clinical and histological categories*, we added these findings that “TBRmax correlated positively with semiquantitative histopathological tumor burden (Spearman ρ =

0.58, $p < 0.001$) and showed a significant increase in PET signal from infiltration zone to solid tumor ($p < 0.001$).” Furthermore, we reflected this relationship in the Discussion, where we now note that “TBRmax was positively associated with semiquantitative histopathological tumor burden and showed a significant stepwise increase across the categories no tumor, infiltration zone, and solid tumor.” Importantly, these findings are consistent with our detection analysis, in which [^{18}F]FET PET demonstrated higher detection rates in solid tumor compared to the infiltration zone (99.1% vs. 82.3%, $p < 0.001$), while maintaining superior detection compared to CE-MRI in both strata.

Taken together, these complementary analyses convincingly indicate that FET-PET signal intensity and detection performance scale are associated with histopathological tumor burden.

2) Would have liked to see data that presents the distance of the evaluated histologic specimens from the closest contrast enhancing disease. Because RT is generally targeted to contrast enhancing disease with some level of margin, demonstrating that FET-PET also performs well further away from contrast enhancing disease will increase confidence that this study will identify infiltrative disease more distant from that identified by CE-MRI.

We thank the reviewer for this thoughtful comment and agree that the distance of each histologic specimen to the nearest contrast-enhancing lesion would be of considerable clinical interest, particularly in the context of radiotherapy target definition. However, we did not perform a specimen-wise distance analysis in the present study. Given the irregular three-dimensional geometry of both the contrast-enhancing lesion and the biopsy targets, a reliable determination of the shortest distance for each individual sample would have required comprehensive slice-by-slice 3D measurements for all specimens, which was beyond the scope of the current work. Nevertheless, because we agree that the spatial relationship between [^{18}F]FET-positivity and contrast-enhancing disease is clinically important, we addressed this issue at the patient level by assessing the extent to which [^{18}F]FET-positivity extended beyond CE-MRI. We have now clarified this point in the Discussion in the radiotherapy section. By explicitly relating it to the findings of our study that are most relevant to this question, we added the following statement: “While we did not perform a specimen-wise distance analysis to the nearest contrast-enhancing lesion, we specifically addressed this issue at the patient level by assessing the spatial extent of [^{18}F]FET-positivity beyond CE-MRI. In this analysis, [^{18}F]FET-positivity frequently extended more than 1 cm beyond CE on MRI with the corresponding [^{18}F]FET+/CE- area showing a very high PPV for viable tumor, as outlined above. Thus, our findings support the relevance of [^{18}F]FET PET for target definition beyond delineation by CE-MRI alone, even though this does not by itself establish a clinical benefit.” This addition was intended to reflect the reviewer’s clinically important point while avoiding overinterpretation beyond the scope of our data.

3) Another assessment that can get at this above question would be to see how well FET-PET performed in the 5 pts where there was no contrast enhancing disease present. If performance was similar to the group as a whole, that would provide greater assurance that FET-PET will perform similarly even when it is more distant from contrast enhancing disease.

We thank the Reviewer for this important suggestion. We have addressed this point by performing an exploratory subgroup analysis of the five patients without contrast enhancement on MRI. All five CE-negative tumors were [^{18}F]FET-positive at the patient level. At the biopsy level, all 30 area-B samples (CE-/[^{18}F]FET+) were histologically tumor-positive, corresponding to a PPV of 100%. This was comparable to the PPV of area B in the overall cohort (98.3%), with no significant difference versus the remainder of the cohort (30/30 vs. 141/144; Fisher’s exact $p = 1.00$). These findings support that [^{18}F]FET PET retains a high positive predictive performance even in the absence of contrast enhancement. Also, in line with the overall cohort [^{18}F]FET-negativity did not reliably exclude tumor in

this subgroup, with an NPV of 26.7% (4/15) among area-D samples (CE-/[^{18}F]FET-). In addition, the PPV of area D1 (CE-/[^{18}F]FET−/FLAIR+) was significantly lower than that of area B (75.0% (9/12) vs. 100% (30/30); Fisher's exact p = 0.019). Importantly, these area-D findings within our subgroup should be interpreted cautiously, as all 11 tumor-positive area-D samples, including all nine tumor-positive D1 samples, originated from a single patient and may therefore be influenced by within-patient clustering. Thus, while [^{18}F]FET-negativity alone was not sufficient to rule out tumor, positive [^{18}F]FET uptake remained highly reliable for predicting histologically confirmed tumor in non-enhancing glioblastoma.

4) (minor) Would like to see another table showing sensitivity/specificity/PPV/NPV of + imaging tests (CE+ and/or FET+, or pooling of groups A-C) vs – imaging tests (CE- and FET-, or group D alone) to see performance when using these as complementary imaging tests. I know this can be derived from the data that is presented but would lay this out more easily for the reader.

We thank the Reviewer for this helpful suggestion. To address this point, we have added a new table, now Table 6, reporting sensitivity, specificity, PPV and NPV for CE-MRI, [^{18}F]FET PET, and their combined use. The combined definition of imaging positivity, CE+ and/or [^{18}F]FET+ corresponding to groups A-C, yielded the highest sensitivity for detecting histopathology-verified tumor (94.4% vs. 55.7% for CE-MRI and 91.2% for [^{18}F]FET PET alone) and maintained a high PPV of 95.1%, compared with 93.1% for CE-MRI and 97.1% for [^{18}F]FET PET alone. The lower specificity of the combined definition is expected, as an "any-positive" approach classifies a sample as positive if either modality is positive, thereby increasing tumor detection at the cost of additional false-positive findings. We have also added a corresponding reference in the Results section to guide the reader to these data more directly.

Imaging test definition	Sensitivity	Specificity	PPV	NPV
**CE+**	55.7%	73.0%	93.1%	20.3%
**[^{18}F]FET+**	91.2%	82.5%	97.1%	59.1%
**CE+ and/or [^{18}F]FET+**	94.4%	68.3%	95.1%	65.2%

Reviewer #3 (Remarks to the Author):

Study summary:
This is a well-executed study addressing the challenging management paradigm of glioblastoma. The core finding - that [18F]FET PET detects viable tumor beyond the contrast-enhancing margin with very high positive predictive value - is supported by the largest histopathologically validated dataset in this context to date. The study is methodologically rigorous with a homogenous and well defined cohort (IDH-wildtype GBM only, classified by 2021 WHO criteria).

We thank the reviewer for the acknowledgment.

A major limitation to the overall relevance of the study is the clinical implications of FET PET positivity in treatment paradigm of GBM noting that recent results from the GLIAA trial did not demonstrate outcome advantage in FET PET vs CE MRI in reirradiation of recurrent GBM.

We thank the reviewer for this important point and agree that the recently published GLIAA trial is highly relevant when discussing the role of [^{18}F]FET PET in the recurrence setting. However, the GLIAA findings need to be interpreted cautiously within the specific context of re-irradiation for recurrent glioblastoma. The GLIAA trial compared two delineation strategies within a re-irradiation paradigm, but it does not answer the more fundamental question of whether re-irradiation itself confers a survival benefit, which still needs to be answered and will be investigated in the ongoing LEGATO trial of the EORTC. Therefore, a negative result of PET-only vs. CE-MRI-only target delineation in recurrent disease should not be overinterpreted as evidence against the broader clinical relevance of [^{18}F]FET PET in glioblastoma in general. Apart from this very important aspect, several other points warrant careful interpretation of the GLIAA data: As mentioned, the GLIAA trial compared [^{18}F]FET PET alone with CE-MRI alone, whereas the current clinical rationale for amino-acid PET is primarily as a complementary modality that adds information beyond MRI rather than replacing it. Accordingly, a comparison of PET+MRI vs. MRI alone would have been conceptually closer to current neuro-oncological practice. Another important point is that the target volumes in the GLIAA trial were overall quite similar between groups despite the well-described spatial mismatch between amino-acid PET and MRI in the literature. This may in part reflect the study design, including the restriction to lesions measuring 1-6 cm, which may have introduced a selection bias and limited the extent of PET-MRI-mismatch captured in the trial. Furthermore, a blinding to the MRI / PET data was not secured, which means that radiation oncologist had access to both imaging types before treatment planning. In such a trial comparing imaging modalities as basis for a local treatment, blinding is crucial and should include technical solutions securing that the other images cannot be seen by the person planning the treatment. Although the GLIAA trial included a formal sample size calculation, this calculation was based on a rather ambitious assumed effect size. The study was therefore likely powered to detect a relatively large benefit of [^{18}F]FET PET -based target delineation alone, but not necessarily smaller yet potentially clinically meaningful effects. In this context, the negative result mainly argues against a large advantage, rather than definitively excluding a more modest benefit. This interpretation is further supported by the use of a one-sided alpha level of 10% in the study. We would also note that GLIAA was conducted in a cohort based on older WHO classifications, which differs from the more homogeneous, molecularly defined WHO 2021 framework used in our study and may further limit direct comparability to contemporary IDH-wildtype glioblastoma cohorts.

Overall, we would therefore caution against interpreting the GLIAA trial as evidence that [^{18}F]FET PET lacks clinical relevance in glioblastoma. By contrast, our histopathology-validated findings remain highly relevant for patient management beyond re-irradiation planning, including biopsy & resection planning, postoperative assessment of residual biologically active tumor, and radiotherapy target definition more broadly.

Major Comments

Spatial misregistration / brain shift

More information required on coregistration between mri and intraop navigation system. What is margin of error . How large are sample sizes ? The acknowledgment of intraoperative brain shift as an inherent limitation is insufficient without an attempt at estimating the spatial error it introduces, since the scientific value of this study rests entirely on the accuracy between preoperative imaging targets and actual tissue sampling locations. This matters most at the boundary between area types, where a 2–3 mm displacement could reclassify a sample area. The authors should report the navigation system's stated targeting accuracy, document any intraoperative accuracy checks performed, and present a sensitivity analysis restricted to stereotactic biopsy cases where spatial

accuracy is highest / easier to maintain and open resection cases. Did the open resection cases start with stereotactic biopsy which was then converted to resection ?

Reproducibility of methodology

Further details regarding methodology is needed to make this a reproducible study. The authors did not specify the size of each biopsy sample acquired.

We thank the reviewer for this important comment. We agree that the spatial accuracy of image-to-tissue correlation is highly relevant for the interpretation of our findings, particularly at the margins between imaging-defined area types. In response, we have substantially expanded the Methods section to provide a more reproducible description of neuronavigation, targeting accuracy, intraoperative accuracy control, timing of tissue sampling, and biopsy specimen size. The following paragraph has been added to the revised manuscript:

"Neuronavigation was performed using the Brainlab Curve navigation system (Cranial Navigation 3.1, Cranial Navigation 4.1, and Cranial Registration 3.6 software; Brainlab SE, Munich, Germany) for both tissue sampling and tumor resection. When applying surface-based registration, the system provides a mean localization error of ≤2.0 mm and a mean trajectory angle error of ≤2°. In frame-based stereotactic procedures, distance and angular measurements have an accuracy of ±0.05 mm and ±0.05°, respectively. Intraoperative accuracy was verified in all open procedures using anatomical landmarks. To minimize the impact of intraoperative brain shift, tissue sampling for study purposes was preferentially performed prior to tumor resection. In cases involving deep-seated targets, sampling was conducted during tumor resection following careful confirmation of navigational accuracy using anatomical landmarks. Targeted intraoperative tissue sampling was guided by neuronavigation and performed under microscopic visualization using fine grasping forceps, yielding specimens with an approximate volume of 3–6 mm^3."

In addition, as suggested, we performed an exploratory biopsy-level subgroup analysis comparing stereotactic biopsy procedures and open resection procedures. Across these two sampling approaches, we found no consistent indication of inferior diagnostic performance in the resection subgroup for either [^{18}F]FET PET, CE-MRI or T2/FLAIR. We also added a brief statement to the Discussion noting that this subgroup analysis did not show reduced diagnostic performance in the resection subgroup, despite this representing the more critical setting with potentially greater spatial inaccuracy than stereotactic biopsy procedures. Exact diagnostic performance metrics for both subgroups are now provided in Supplementary Table 1.

While it is apparent that multiple samples were taken from each patient, there is no detailed explanation. Did one patient have 10 samples while another had 2? Please show this as part of table 1.

To improve transparency regarding the sampling distribution across patients, we have now added the number of biopsies per patient to Table 1 as a summary measure (median and range) for easier reference.

What software was used for co-registration? What software version number etc.

We thank the Reviewer for this important comment. We have now clarified the software used for both components of the image coregistration workflow in the revised Methods section. Image-guided coregistration for intraoperative neuronavigation was performed using the Brainlab Curve navigation system with Cranial Navigation 3.1, Cranial Navigation 4.1, and Cranial Registration 3.6 software (Brainlab SE, Munich, Germany). In addition, image analysis and spatial correlation of the sampling coordinates with the corresponding imaging datasets were performed on a Hermes workstation using Hermes' HERMIA Hybrid Viewer PDR version 6.1.4 (Hermes Medical Solutions, Stockholm, Sweden).

Specifically, the stereotactic CT or MRI datasets containing the marked sampling coordinates were re-imported together with the coregistered [^{18}F]FET PET, contrast-enhanced T1-weighted MRI, and T2/FLAIR datasets. The sampling coordinates were then projected across the coregistered datasets using the "Triangulate" function, and PET ROIs were placed with the integrated "Quick ROI" tool. These software details, including version numbers, have been added to the Methods section to improve reproducibility.

Clarification on definition of solid tumor - histopathology

What do the authors mean by "in most cases" there is microvascular proliferation and/or necrosis. What were the exact histopathologic definition? What about mitotic figures/high mitotic rate (how many per high power field)? Are there cases included that did not have these features? If so, then are these defined based on molecular features?
"Tumor cells formed confluent, pleomorphic proliferations with frequent mitoses and, in most cases, microvascular proliferation and/or necrosis."

We appreciate the Reviewer's careful reading and agree that the previous wording did not sufficiently distinguish the biopsy-level definition of "solid tumor" from histologic WHO grade 4 criteria, particularly with regard to the phrase "in most cases, microvascular proliferation and/or necrosis" and the non-quantified description of "frequent mitoses. We have therefore revised the Methods section to more clearly define "solid tumor" at the biopsy-sample level and to separate this definition from the mandatory presence of individual WHO grade 4 features. The revised definition now reads:

"Solid tumor: Densely cellular glioblastoma with near-complete effacement of normal cortical or white-matter architecture and only rare residual neurons or glial cells of non-neoplastic morphology. Tumor cells formed confluent, pleomorphic proliferations with variable mitotic activity. Microvascular proliferation and/or necrosis were common but not required for the biopsy-level definition of solid tumor, which was based on compact tumor-cell growth and replacement of normal brain parenchyma."

To further address the Reviewer's question, we performed a patient-level review of the neuropathology reports. All 61 patients had an integrated diagnosis of glioblastoma, IDH-wildtype, CNS WHO grade 4 according to the 2021 WHO Classification. Histologic WHO grade 4 features, defined as microvascular proliferation and/or necrosis, were documented in 52 of 61 patients (85.2%). In 9 of 61 patients (14.8%), neither microvascular proliferation nor necrosis was documented in the available reports. Among these, all 9 patients had documented WHO-defined molecular features of glioblastoma, IDH-wildtype, most commonly TERT promoter mutation.

We also agree that mitotic activity was not sufficiently defined in the previous wording. Mitotic activity was documented heterogeneously in the routine neuropathology reports, with quantitative mitotic counts available only in a minority of cases. Therefore, mitotic activity was not used as a separate quantitative study variable, and no minimum mitotic count per high-power field was required for classification as solid tumor. We have revised the Methods section accordingly and replaced the ambiguous wording.

FLAIR+ signal as a segmentation

While the authors subdivided areas A-D by FLAIR signal, they did not evaluate FLAIR+ as a primary comparison to FET positive. In addition to contrast enhancement, FLAIR+ signal change is also used as

a primary driver to evaluate for disease infiltration particularly among patients with recurrence. Consider adding this to the discussion section.

We thank the reviewer for this important comment and agree that T2/FLAIR signal alterations play a key role in the assessment of tumor infiltration, particularly in the recurrent setting. While our study stratified biopsy locations according to FLAIR signal, a direct comparison between FLAIR-positive and [^{18}F]FET-positive tissue was not defined as a primary objective and cannot be robustly addressed based on the present dataset. We have therefore clarified this limitation in the Discussion. The following sentence has been added:

"… the absence of systematic sampling in FLAIR+/[^{18}F]FET- tissue further limits conclusions regarding the primary comparison of these imaging modalities, particularly in recurrent disease, where T2/FLAIR-positivity is a main determinant for detection of disease infiltration."

Sampling bias and under-sampling of FET-negative cases (Area C and D)
Area C contains only 22 samples, yielding a PPV of 59.1%. This under-sampling reflects the study's deliberate focus on FET-positive regions, but it leaves unresolved a clinically important question: whether PET negativity in contrast-enhancing tissue reliably identifies treatment effect, radiation necrosis, or non-tumoral blood-brain barrier disruption, particularly in the post-treatment setting. The authors should report confidence intervals in table 5 and address this shortcoming in the discussion section.

We thank the reviewer for this important comment. As suggested, we have added 95% confidence intervals to Table 5 and updated the corresponding table legend accordingly. In addition, we have expanded the Discussion (limitations section) to more comprehensively address the under-sampling of [^{18}F]FET-negative regions (areas C and D) and the resulting limitations in interpreting PET-negative findings. We now explicitly address, that this affects the assessment of PET-negativity in contrast-enhancing tissue in the post-treatment setting. The following sentence has been added:

"This limits conclusions regarding the extent to which PET-negativity reflects tumor-negative tissue in general and, in the presence of contrast enhancement, reliably indicates non-tumoral blood-brain barrier disruption, such as radiation necrosis in the post-treatment setting (…)."

The recurrence subgroup is underpowered and Area D within recurrence is not obtained
The recurrence cohort of 17 patients and 95 samples is underpowered. Additionally, the absence of any Area D biopsies in this group means the B vs D or A vs D comparisons where distinguishing viable tumors from treatment-related change is the central diagnostic question cannot be performed at all. The reported PPV values for Areas A and B in recurrent disease are therefore derived from a highly selected, PET-positive sample that cannot address whether FET-negative, non-enhancing tissue in this context is truly tumor-free or simply below the tracer's detection threshold after treatment-induced microstructural changes. Table 2 is confusing to have both initial diagnosis and recurrent cases (in particular area D cohort - as it does not include any recurrent cases). Consider splitting initial and recurrent cases as the clinical question for recurrence often hinges on FLAIR changes which is not as important for initial diagnosis.

We thank the reviewer for this thoughtful comment and agree that the recurrence subgroup is limited in size and that the absence of CE-/[^{18}F]FET- biopsies (area D) restricts the interpretation of our findings in this regard. As correctly pointed out, this prevents a direct assessment of whether PET-negative, non-enhancing tissue in recurrent disease is truly tumor-free or reflects tumor below the detection threshold. It also means that comparisons such as area A vs. D or B vs. D, which would be required to directly address the distinction between viable tumor and treatment-related change in this subgroup,

could not be performed. We agree that this is a clinically important question, particularly in the post-treatment setting. However, the absence of area D samples in the recurrent subgroup reflects the clinically indicated sampling strategy, in which biopsies were preferentially obtained from imaging-suspicious regions, whereas CE-/[^{18}F]FET- tissue was only rarely sampled due to limited rationale. We have explicitly addressed this limitation in the Discussion with the following sentence:

"This limits conclusions regarding the extent to which PET-negativity reflects tumor-negative tissue in general and, in the presence of contrast enhancement, reliably indicates non-tumoral blood-brain barrier disruption, such as radiation necrosis in the post-treatment setting, rather than tumor below the tracer's detection threshold. This limitation is especially relevant in the recurrent subgroup, which was small and did not include any CE-/[^{18}F]FET- samples (area D). Accordingly, diagnostic performance measures in the post-treatment setting should be interpreted with caution (…)"

In addition, to improve clarity and better reflect the different clinical contexts, we have split Table 2 into separate tables for initial diagnosis and recurrent disease, now provided as Supplementary Tables S2A and S2B. As now stated in the Results section, "The same distribution is additionally provided separately for the initial diagnosis and recurrence cohorts in Supplementary Tables S2A and S2B."

Please explain results of table 6: under recurrence group - Area A specificity = 22.2 vs Area B specificity = 100.0

We thank the reviewer for pointing out this apparent discrepancy. The marked difference in specificity between Area A (22.2%) and Area B (100.0%) in the recurrence subgroup is primarily explained by the very small number of histologically tumor-negative samples in this subgroup. Specifically, only 9 tumor-negative samples were available in the recurrence cohort. Of these, 7 were located in Area A, resulting in a relatively high number of false-positive findings and consequently a low specificity (2/9 = 22.2%). In contrast, no tumor-negative samples were observed in Area B (0 false positives), yielding a specificity of 100% (9/9 true negatives). Thus, the observed difference is driven by the distribution of a very limited number of negative cases in the setting of recurrence.

REVIEWERS' COMMENTS

Reviewer #1 (Remarks to the Author):

The authors have adequately addressed all the concerns raised in my previous review. I have no further comments and consider the revised manuscript suitable for publication in its current form.

We thank the reviewer for the positive assessment of our revised manuscript.

Reviewer #2 (Remarks to the Author):

This manuscript prospectively enrolled patients on a study where histopathological assessment was spatially linked to FET-PET and MRI scans in WHO grade 4 glioblastomas (w/IDH wild type). They examined 472 specimens from 61 patients where each specimen was spatially linked with corresponding pre-operative FET-PET and MRI scans with each being labeled as CE+/FET+ (A), CE-/FET+ (B), CE+/FET- (C) and CE-/FET- (D) with further classification without (0) or with (1) FLAIR signal. FET was considered + if value was >1.6-fold the mean background activity. They found that FET-PET showed greater sensitivity, specificity, PPV and NPV for detecting presence of tumor than CE- or FLAIR-MRI.

I had several critiques in my initial review that the authors thoughtfully addressed. I do not have additional suggestions at this time.

We thank the reviewer for their positive evaluation and for the constructive feedback provided throughout the review process.

Reviewer #3 (Remarks to the Author):

I thank the authors for their thorough and thoughtful revision of the manuscript. The rebuttal letter adequately addresses each of the concerns raised in the initial review. The additional analyses/clarifications strengthen the scientific rigor and clarity of the manuscript, and the revised text improves the overall readability and impact of the work. I have no further concerns and believe the manuscript is suitable for publication in its current form.

We appreciate the reviewer's favorable assessment of the revised manuscript and their helpful comments, which contributed to improving the manuscript.

Reviewer #4 (Remarks to the Author):

I co-reviewed this manuscript with one of the reviewers who provided the listed reports. This is part of the Nature Communications initiative to facilitate training in peer review and to provide appropriate recognition for Early Career Researchers who co-review manuscripts.

We thank the reviewer for their contribution to the review process.
